# Time to Full Enteral Feeds and Late-Onset Sepsis in Extremely Preterm Infants

**DOI:** 10.1001/jamanetworkopen.2025.43940

**Published:** 2025-11-17

**Authors:** Ariel A. Salas, Laura Elizabeth Wiener, Marissa Trotta, Vivian Valcarce, Mar Romero-Lopez, Eric B. Ortigoza, Ting Ting Fu, Kera McNelis, Brenda Poindexter, Waldemar A. Carlo

**Affiliations:** 1 Division of Neonatology, Department of Pediatrics, University of Alabama at Birmingham, Birmingham; 2 Biostatistics and Epidemiology Division, RTI International, Research Triangle Park, North Carolina; 3 Division of Perinatal-Neonatal Medicine, Department of Pediatrics, University of Texas Health Science Center Houston, Houston; 4 Division of Neonatal-Perinatal Medicine, Department of Pediatrics, UT Southwestern Medical Center, Dallas, Texas; 5 Perinatal Institute, Division of Neonatology, Cincinnati Children’s Hospital Medical Center, Cincinnati, Ohio; 6 Department of Pediatrics, University of Cincinnati College of Medicine, Cincinnati, Ohio; 7 Division of Neonatology, Department of Pediatrics, Children’s Healthcare of Atlanta and Emory University, Atlanta, Georgia

## Abstract

**Importance:**

Recent studies suggest that early achievement of full enteral feeding improves clinical outcomes among preterm infants.

**Objective:**

To examine the association between full enteral feeding and late-onset sepsis.

**Design, Setting, and Participants:**

This secondary analysis of a cohort study prospectively followed up a multicenter cohort of preterm infants with gestational ages ranging from 23 to 28 weeks born between January 1, 2012, and December 31, 2021, at 19 US academic centers. Infants without major anomalies who received enteral feedings and survived beyond postnatal day 7 were included.

**Exposure:**

Full enteral feeding (≥120 mL/kg/d).

**Main Outcomes and Measures:**

The primary outcome was the incidence of late-onset sepsis confirmed through culture-positive results occurring more than 72 hours after birth and treated with antibiotics for 5 or more days. Other clinical outcomes assessed up to 36 weeks of postmenstrual age included necrotizing enterocolitis, death, and growth faltering (weight, length, or head circumference *z* score decrease >1.2). Risk estimates were adjusted for clinical variables associated with acute critical illness and birth year.

**Results:**

Demographic and clinical data from 15 102 preterm infants were analyzed (mean [SD] maternal age, 28.7 [6.1] years; mean [SD] gestational age, 26.0 [1.6] weeks; mean [SD] birth weight, 875 [242] g; 7648 male [50.6%]). Between January 1, 2012, and December 31, 2021, the median (IQR) time to achieve full enteral feeding decreased from 18 (14-28) days to 14 (10-22) days, and the incidence of late-onset sepsis decreased from 21.1% to 16.5% (*P* = .003). In adjusted analyses, the relative risk of late-onset sepsis per each additional 1-week delay in achieving full enteral feeding was 16% higher (adjusted relative risk [ARR], 1.16; 95% CI, 1.14-1.18; *P* < .001). Delays in achieving full enteral feeding were also associated with a higher risk of necrotizing enterocolitis (ARR, 1.20; 95% CI, 1.16-1.24; *P* < .001) and growth faltering in weight (ARR, 1.08; 95% CI, 1.07-1.09), length (ARR, 1.03; 95% CI, 1.02-1.03), and head circumference (ARR, 1.07; 95% CI, 1.06-1.08; *P* < .001 for all).

**Conclusions and Relevance:**

In this cohort study of preterm infants with gestational ages ranging from 23 to 28 weeks who received enteral feeds and survived beyond postnatal day 7, delays in establishing full enteral feeding were associated with a higher risk of late-onset sepsis. These results suggest that early initiation and advancement of enteral feeding have the potential to reduce the risk of late-onset sepsis, growth faltering, and necrotizing enterocolitis.

## Key Points

**Question:**

Is there an association between time to full enteral feeding and late-onset sepsis in extremely preterm infants?

**Findings:**

In this cohort study of 15 102 extremely preterm infants, each additional 1-week delay in achieving full enteral feeding was associated with a 16% higher relative risk of late-onset sepsis in adjusted analyses.

**Meaning:**

These results suggest that delays in establishing full enteral feeding are associated with a higher risk of late-onset sepsis.

## Teaser

This cohort study of extremely preterm infants evaluates whether there is an association between full enteral feeding and late-onset sepsis, necrotizing enterocolitis, and growth faltering.

## Introduction

Infants born extremely preterm (EPT) face a complex and multifaceted journey, particularly during the first 2 weeks after birth in which acute critical illness is most severe^1^ and establishing early and adequate enteral nutrition is challenging.^2^ Historically, clinicians have been cautious about initiating enteral feeding in EPT infants due to concerns about gastrointestinal immaturity and the potential risk of severe complications, particularly necrotizing enterocolitis (NEC).^3^ As a result, enteral fasting or prolonged minimal enteral feeding were common practices for years.^4^

In recent years, advances in neonatal care have transformed feeding practices. Mothers are now encouraged to express and provide their milk as early as possible,^5^ and when maternal milk is not available, donor milk feeding is prioritized. This shift has allowed many clinicians worldwide to implement strategies aimed at achieving full enteral feeding within days of birth, even for the most at-risk EPT infants.^6,7^ Human milk feeding promotes gut colonization with beneficial bacteria,^8^ supports immune system development, and reduces the risk of late-onset sepsis (LOS).^7,9,10^ An increasing body of evidence has highlighted the advantages of early achievement of full enteral feeding. Notably, contrary to longstanding concerns, early enteral feeding with human milk has not been associated with a higher risk of NEC in meta-analyses of randomized clinical trials of daily advancement of enteral feeding volumes before or on postnatal day 4 (early progression of enteral feeding)^11^ and rapid advancement of enteral feeding volumes by 30 to 40 mL/kg/d (faster progression of enteral feeding).^12^

Despite the widespread recognition of these benefits, reports of delays in establishing full enteral feeding during the acute phase of critical illness remain common in neonatal intensive care units (NICUs).^1,13^ This inconsistency highlights the need for well-designed multicenter studies that characterize potential benefits and harms associated with the early establishment of full enteral feeding. This study examined the association between full enteral feeding and LOS, NEC, and growth faltering in EPT infants.

## Methods

This study was a secondary analysis of a prospective cohort study of infants with gestational ages ranging from 23 to 28 weeks born between January 1, 2012, and December 31, 2021. Using a prospective registry from the Eunice Kennedy Shriver National Institute of Child Health and Human Development Neonatal Research Network (NRN)^14^ that includes academic NICUs with access to comprehensive obstetric care and the highest level of neonatal care available, we identified EPT infants without major congenital or chromosomal anomalies who received enteral feedings and survived beyond postnatal day 7. The institutional review board for each participating institution approved participation in the registry. Waiver of consent for enrollment in the registry was granted for most institutions. In institutions where waiver of consent was not approved, written informed consent was obtained from parents or guardians before enrollment in the registry. The report of this study follows the Strengthening the Reporting of Observational Studies in Epidemiology (STROBE) reporting guideline for cohort studies.

A flow diagram of the study population is shown in eFigure in Supplement 1. From 2012 to 2021, a total of 20 944 infants born between 22 and 29 weeks of gestation were admitted to NRN centers. Of 15 373 infants who met inclusion criteria, 15 102 had sufficient data available for analysis and 11 349 (75.1%) met the World Health Organization definition of extreme prematurity.

The primary exposure of interest, full enteral feeding, was defined as a volume intake of 120 mL/kg/d or more. This threshold, routinely captured in our registry, was selected because most participating NICUs use it as a criterion for discontinuing parenteral nutrition. In addition to calculating the time required to achieve full enteral feeding, we estimated the total number of full enteral feeding days in the first 28 days after birth, a surrogate measure of feeding tolerance that can be assessed in all study participants and ranged from 0 to 28 days. With this information, we generated risk estimates for the association between early enteral feeding and various outcomes. We also estimated the percentage of infants who achieved full enteral feeding at different postnatal weeks and day intervals during the first 28 days after birth.

The primary outcome of LOS was defined as a positive blood culture occurring more than 72 hours after birth and treated with antibiotics for 5 or more days, independent of clinical signs of sepsis. We specifically focused on the first episode of LOS and routinely collected data on pathogens.

Secondary outcomes were mortality, spontaneous intestinal perforation without NEC, any stage 2 or 3 NEC (per modified Bell staging), postnatal growth faltering at 36 weeks of postmenstrual age (PMA) or discharge (defined as a decrease in Fenton weight *z* score^15^ from birth to 36 weeks’ PMA or discharge >1.2),^16^ duration of parenteral nutrition, and length of hospital stay.

### Sample Size

In our pilot trial in which more than 80% of infants achieved full enteral feeding within the first 2 weeks after birth, 18% of EPT infants developed LOS (10% in the early feeding progression group and 27% in the delayed feeding progression group; *P* = .18).^7^ Culture-proven sepsis or death occurred in 28% of study participants (nonsignificant risk difference favoring the early feeding group: 4%). Thus, we anticipated that a sample size of 3142 patients (786 infants exposed to early achievement of full enteral feeding and 2356 infants unexposed) would have a 90% power to detect a 6% absolute risk reduction in culture-proven sepsis or death at a *P* < .05 significance level. This estimated risk reduction falls within the range reported in meta-analyses of clinical trials on early feeding practices (2%-10%)^11,12^ and in observational studies from developed countries (6%-10%).^18,19,20^

### Statistical Analysis

We performed descriptive statistics and risk-adjusted modeling. Continuous variables were summarized using mean (SD) for normally distributed data or median (IQR) for skewed distributions. Categorical variables were described as numbers (percentages). The annual proportions of infants who achieved full enteral feeding within the first 10 days after birth or experienced postnatal growth faltering, LOS, NEC, and mortality were graphed for the study period. Differences between 2012 and 2021 were compared using Cochran-Mantel-Haenszel tests, controlling for NRN center.

To estimate the association between enteral feeding and the incidence of LOS, we used robust Poisson regression models. Covariates were selected a priori based on clinical relevance and included NRN center as a fixed effect, 5-minute Apgar score, small for gestational age status, maternal characteristics (education level, socioeconomic status, type of insurance, and pregnancy complications, such as hypertension or diabetes), birth year to account for evolving clinical practices, and the probability of survival without bronchopulmonary dysplasia (BPD) at birth and at postnatal day 7 in a continuous scale as a proxy of severity of critical illness. We used the BPD estimate calculator to determine the probability of survival without BPD based on gestational age, birth weight, sex, maternal race, type of ventilator support, and fraction of inspired oxygen.^17^ Maternal race categories included American Indian or Alaska Native, Asian, Black, Native Hawaiian or Pacific Islander, White, and other (>1 race or unknown racial and ethnic subcategories). Data on self-reported maternal race were collected because race is one of the parameters required to estimate the probability of survival without BPD using the BPD estimate calculator. The probability of survival without BPD at birth and the change in the probability of survival without BPD from birth to postnatal day 7 were both included in the adjusted models.

Full enteral feeding was analyzed as a continuous exposure (ie, number of days from birth to full enteral feeding and total number of days receiving full enteral feeding during the first 28 days) and as an ordinal exposure at fixed intervals (time to full feeding at different postnatal weeks and days). All analyses were conducted using SAS software, version 9.4 (SAS Institute Inc) with a 2-sided significance level of *P* < .05.

## Results

Demographic and clinical data from 15 102 preterm infants were analyzed (mean [SD] maternal age, 28.7 [6.1] years; 151 [1.0%] American Indian or Alaska Native, 582 [3.9%] Asian, 5962 [39.6%] Black, 75 [0.5%] Native Hawaiian or Pacific Islander, 7641 [50.7%] White, and 657 [5.3%] other; mean [SD] gestational age, 26.0 [1.6] weeks; mean [SD] birthweight, 875 [242] g; 7648 male [50.6%] and 7454 female [49.4%]). Maternal and neonatal characteristics by postnatal week in which full enteral feeding was achieved are given in Table 1. Baseline characteristics of infants who did not achieve full enteral feeding by 36 weeks’ PMA are also summarized in Table 1.

**Table 1.** Maternal and Neonatal Characteristics by Postnatal Week in Which Full Enteral Feeding Was Achieved

Characteristic	No. (%) of mothers or infants							
	Full enteral feeding achieved by 36 weeks’ PMA						Did not achieve full feeds (n = 1046)	Overall (N = 15 102)
	Week 1 (n = 292)	Week 2 (n = 5396)	Week 3 (n = 3881)	Week 4 (n = 1788)	>Week 4 (n = 2699)	Overall (n = 14 056)		
**Maternal characteristics**								
Age, mean (SD), y	28.8 (6.2)	28.8 (6.1)	29.0 (6.2)	28.6 (6.2)	28.7 (6.1)	28.8 (6.1)	28.0 (6.1)	28.7 (6.1)
Married	133 (45.5)	2348 (43.8)	1799 (46.6)	803 (45.1)	1213 (45.1)	6296 (45.0)	461 (44.5)	6757 (45.0)
College degree or partial college	106 (36.3)	1984 (36.8)	1412 (36.4)	651 (36.4)	1053 (39.0)	5206 (37.0)	379 (36.2)	5585 (37.0)
Public insurance status	114 (39.3)	2017 (37.5)	1555 (40.2)	706 (39.7)	1068 (39.7)	5460 (39.0)	403 (38.6)	5863 (38.9)
Hispanic or Latino ethnicity	46 (15.8)	925 (17.3)	552 (14.4)	278 (15.8)	396 (14.9)	2197 (15.8)	140 (13.6)	2337 (15.7)
Race^a^								
American Indian or Alaska Native	2 (0.7)	54 (1.0)	47 (1.2)	15 (0.8)	24 (0.9)	142 (1.0)	9 (0.9)	151 (1.0)
Asian	13 (4.5)	190 (3.5)	163 (4.2)	72 (4.0)	101 (3.7)	539 (3.8)	43 (4.1)	582 (3.9)
Black	111 (38.0)	2168 (40.3)	1482 (38.3)	702 (39.4)	1089 (40.4)	5552 (39.6)	410 (39.3)	5962 (39.6)
Native Hawaiian or Pacific Islander	0 (0.0)	28 (0.5)	16 (0.4)	12 (0.7)	13 (0.5)	69 (0.5)	6 (0.6)	75 (0.5)
White	162 (55.5)	2716 (50.4)	2007 (51.8)	895 (50.2)	1337 (49.6)	7117 (50.7)	524 (50.2)	7641 (50.7)
Other^b^	4 (1.3)	229 (4.3)	156 (4.1)	87 (4.9)	130 (4.8)	606 (4.3)	51 (4.9)	657 (4.3)
Multiple gestation	77 (26.4)	1341 (24.9)	997 (25.7)	446 (24.9)	726 (26.9)	3587 (25.5)	293 (28.0)	3880 (25.7)
Hypertension								
Pregnancy induced	29 (9.9)	731 (13.5)	670 (17.3)	303 (17.0)	406 (15.1)	2139 (15.2)	141 (13.5)	2280 (15.1)
Chronic	43 (14.7)	763 (14.1)	583 (15.0)	274 (15.4)	400 (14.8)	2063 (14.7)	149 (14.3)	2212 (14.7)
Diabetes, insulin given	13 (4.5)	166 (3.1)	126 (3.3)	63 (3.5)	60 (2.2)	428 (3.1)	36 (3.5)	464 (3.1)
Complete course of antenatal steroids	206 (76.0)	3920 (77.9)	2759 (77.2)	1196 (73.3)	1825 (74.2)	9906 (76.4)	703 (73.9)	10609 (76.2)
**Neonatal characteristics**								
Birth weight, mean (SD), g	1094 (244)	993 (230)	867 (213)	809 (206)	740 (197)	888 (239)	698 (205)	875 (242)
Sex								
Male	156 (53.8)	2731 (50.6)	1863 (48.0)	922 (51.6)	1413 (52.4)	7086 (50.4)	562 (53.7)	7648 (50.6)
Female	135 (46.2)	2665 (49.4)	2018 (52.0)	866 (48.4)	1286 (47.7)	6970 (49.6)	484 (46.3)	7454 (49.4)
Gestational age, wk								
23	6 (2.1)	123 (2.3)	184 (4.7)	141 (7.9)	461 (17.1)	915 (6.5)	276 (26.4)	1191 (7.9)
24	12 (4.1)	301 (5.6)	446 (11.5)	303 (16.9)	598 (22.2)	1660 (11.8)	262 (25.0)	1922 (12.7)
25	11 (3.8)	541 (10.0)	678 (17.5)	397 (22.2)	583 (21.6)	2210 (15.7)	180 (17.2)	2390 (15.8)
26	28 (9.6)	899 (16.7)	850 (21.9)	365 (20.4)	491 (18.2)	2633 (18.7)	142 (13.6)	2775 (18.4)
27	73 (25.0)	1448 (26.8)	847 (21.8)	288 (16.1)	321 (11.9)	2977 (21.2)	94 (9.0)	3071 (20.3)
28	162 (55.5)	2084 (38.6)	876 (22.6)	294 (16.4)	245 (9.1)	3661 (26.0)	92 (8.8)	3753 (24.9)

Abbreviation: PMA, postmenstrual age.

^a^ Race and ethnic groups are reported by parent or guardian.

^b^ More than 1 race or unknown racial and ethnic subcategories.

The median (IQR) time to achieve full enteral feeding was 18 (14-28) days in 2012 and 14 (10-22) days in 2021 (Table 2). LOS was identified in 2736 of 15 102 infants (18.1%). The median (IQR) time from birth to the first episode of LOS was 15 (10-26) days in 2012 and 2021. The most common pathogen identified in blood cultures was coagulase-negative staphylococci (CONS), and the main reduction in LOS over time seemed to be accounted for by a reduction in the treatment of positive blood cultures for CONS (eTable in Supplement 1). Although the absolute incidence of LOS decreased by 4.6 percentage points (from 21.1% [344 of 1630] to 16.5% [235 of 1427]) from 2012 to 2021 (*P* = .003), the proportion of infants who achieved full enteral feeding within the first 10 days after birth increased from 6.6% (100 of 1513) in 2012 to 26.3% (353 of 1343) in 2021 (*P* < .001) (Figure, A). Between 2012 and 2021, NEC frequency remained at approximately 10% (9.4% [153 of 1630] vs 10.4% [148 of 1426]; *P* = .21), the proportion of infants with weight faltering decreased (54.8% [808 of 1474] vs 48.7% [626 of 1285]; *P* = .009), the proportion of infants with head circumference faltering increased (42.3% [602 of 1423] vs 51.1% [638 of 1249]; *P* < .001), and the proportion of infants with length faltering remained unchanged (67.7% [938 of 1386] vs 68.7% [844 of 1229]; *P* = .92) (Figure, B). Mortality after postnatal day 7 also remained unchanged (6.9% [113 of 1626] vs 6.0% [86 of 1426]; *P* = .21).

**Table 2.** Incidence of Pathogens by Birth Year

Birth year	Time to full feeds by 36 wk, median (IQR), d	LOS, No. (%)	Time from birth to first LOS, median (IQR), d	Distribution of pathogens identified during the first episode of LOS, No. (%)										
				CONS	MRSA or MSSA	*Escherichia coli*	*Klebsiella*	*Pseudomonas*	*Serratia*	*Enterobacter*	*Enterococcus*	*Candida*	*Streptococcus *B	Other
2012	18 (14-28)	344 (21.1)	15 (10-26)	178 (49.3)	48 (13.3)	26 (7.2)	15 (4.2)	11 (3)	4 (1.1)	14 (3.9)	11 (3)	19 (5.3)	12 (3.3)	23 (6.4)
2013	18 (14-27)	258 (16.7)	18 (11-29)	139 (50)	48 (17.3)	19 (6.8)	10 (3.6)	10 (3.6)	7 (2.5)	7 (2.5)	7 (2.5)	8 (2.9)	8 (2.9)	15 (5.4)
2014	18 (14-27)	302 (19.5)	16 (9-30)	155 (49.8)	39 (12.5)	31 (10)	15 (4.8)	17 (5.5)	4 (1.3)	6 (1.9)	12 (3.9)	7 (2.3)	8 (2.6)	17 (5.5)
2015	17 (14-27)	307 (19.7)	16 (10-28)	144 (44.7)	58 (18)	33 (10.2)	19 (5.9)	6 (1.9)	7 (2.2)	10 (3.1)	8 (2.5)	12 (3.7)	9 (2.8)	16 (5)
2016	16 (12-24)	259 (18.1)	18 (10-31)	114 (41.6)	48 (17.5)	23 (8.4)	14 (5.1)	8 (2.9)	8 (2.9)	6 (2.2)	16 (5.8)	7 (2.6)	10 (3.6)	20 (7.3)
2017	16 (12-24)	260 (18.1)	15 (9-27)	116 (42.8)	49 (18.1)	30 (11.1)	12 (4.4)	3 (1.1)	6 (2.2)	4 (1.5)	11 (4.1)	14 (5.2)	10 (3.7)	16 (5.9)
2018	15 (11-22)	264 (17.7)	14 (9-25)	106 (38.8)	44 (16.1)	35 (12.8)	24 (8.8)	5 (1.8)	9 (3.3)	9 (3.3)	13 (4.8)	13 (4.8)	7 (2.6)	8 (2.9)
2019	15 (11-23)	272 (17.1)	16 (10-30)	111 (38.5)	46 (16)	36 (12.5)	23 (8)	13 (4.5)	7 (2.4)	7 (2.4)	8 (2.8)	11 (3.8)	12 (4.2)	14 (4.9)
2020	14 (11-23)	235 (16.3)	17 (10-28)	98 (39.4)	45 (18.1)	26 (10.4)	12 (4.8)	10 (4)	9 (3.6)	8 (3.2)	5 (2)	8 (3.2)	11 (4.4)	17 (6.8)
2021	14 (10-22)	235 (16.5)	15 (10-26)	103 (42.2)	42 (17.2)	28 (11.5)	16 (6.6)	6 (2.5)	6 (2.5)	5 (2)	13 (5.3)	5 (2)	6 (2.5)	14 (5.7)

Abbreviations: CONS, coagulase-negative *Staphylococcus*; LOS, late-onset sepsis; MRSA, methicillin-resistant *Staphylococcus aureus*; MSSA, methicillin-sensitive *Staphylococcus aureus*.

**Figure.**
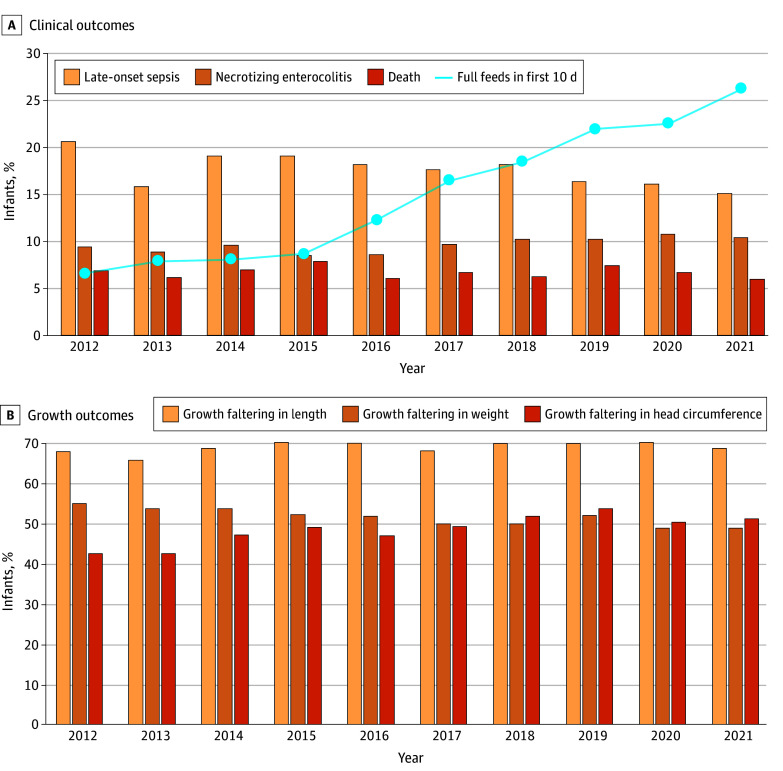
Trends in Early Full Enteral Feeding Within First 10 Days After Birth, Clinical Outcomes, and Growth Outcomes in Infants Born Extremely Preterm

Infants who achieved full enteral feeding within the first week after birth had less critical illness at birth, shorter exposure to parenteral nutrition, reduced length of hospital stays, lower rates of in-hospital morbidities, and smaller decreases in length and head circumference *z* scores from birth to 36 weeks’ PMA than those who reached full feeding at later week intervals (Table 3). The association between time to achieve full enteral feeding in 1-week increments and in-hospital outcomes by 36 weeks’ PMA and between number of days of full enteral feeding in the first 28 days and in-hospital outcomes by 36 weeks’ PMA is shown in Table 4. Each additional 1-week delay in achieving full enteral feeding was associated with a higher adjusted risk of LOS (adjusted relative risk [ARR], 1.16; 95% CI, 1.14-1.18; *P* < .001). Each additional day of full feeding in the first 28 days after birth was associated with a lower risk of LOS (ARR, 0.95; 95% CI, 0.94-0.96; *P* < .001). Delays in achieving full enteral feeding were also associated with a higher risk of NEC (ARR, 1.20; 95% CI, 1.16-1.24; *P* < .001) and growth faltering in weight (ARR, 1.08; 95% CI, 1.07-1.09), length (ARR, 1.03; 95% CI, 1.02-1.03), and head circumference (ARR, 1.07; 95% CI, 1.06-1.08; *P* < .001 for all).

**Table 3.** Nutritional Characteristics and In-Hospital Morbidities by Postnatal Week in Which Full Enteral Feeding Was Achieved

Characteristic	No. (%) of infants							
	Achieved full feeds by 36 weeks’ PMA						Did not achieve full feeds (n = 1046)	Total (N = 15 102)
	Week 1 (n = 292)	Week 2 (n = 5396)	Week 3 (n = 3881)	Week 4 (n = 1788)	Later than week 4 (n = 2699)	Total (n = 14 056)		
**Nutrition**								
Duration of parenteral nutrition, median (IQR), d	7 (6-11)	11 (9-13)	17 (15-21)	25 (22-29)	39 (32-51)	17 (12-29)	19 (11-75)	17 (12-30)
Age at first enteral feed, median (IQR), d^a^	2 (2 to 2)	2 (2-3)	3 (2-4)	3 (2-5)	4 (2-6)	3 (2-4)	4 (2-6)	3 (2-4)
Age at full enteral feeding, median (IQR), d^a^	7 (6-7)	12 (10-13)	17 (16-19)	24 (23-26)	37 (32-46)	16 (12-25)	NA	16 (12-25)
Duration of full enteral feeding by 28 DOLs, median (IQR), d	22 (22-23)	17 (16-19)	12 (10-13)	5 (3-6)	0 (0-0)	13 (4-17)	0 (0-0)	12 (1-17)
Breast milk in the first 28 d	282 (98.9)	5272 (98.9)	3756 (98.5)	1718 (98.0)	2573 (98.1)	13601 (98.5)	982 (97.0)	14583 (98.4)
Weight fell below birth weight in first 10, d^b^	53 (96.4)	1692 (96.4)	1674 (93.2)	794 (91.0)	1185 (89.3)	5398 (93.0)	394 (84.9)	5792 (92.4)
Age when birth weight regained, median (IQR), d	13 (11-17)	11 (8-15)	10 (7-13)	10 (7-13)	10 (7-13)	10 (8-13)	8 (6-11)	10 (7-13)
**Growth^c^**								
Weight								
*z *Score at birth, mean (SD)	0.4 (0.8)	0.1 (0.8)	−0.1 (0.9)	−0.2 (0.9)	−0.3 (1.0)	−0.1 (0.9)	−0.4 (1.1)	−0.1 (0.9)
*z* Score at 36 weeks, mean (SD)	−0.9 (0.9)	−1.1 (0.9)	−1.3 (0.9)	−1.5 (1.0)	−1.8 (1.1)	−1.3 (1.0)	−1.7 (1.2)	−1.33 (1.0)
*z* Score change from birth to 36 wk <−1.2	149 (53.0)	2416 (47.4)	1710 (46.2)	931 (54.6)	1689 (64.3)	6895 (51.4)	197 (56.4)	7092 (51.5)
Less than 3rd percentile at 36 wk	33 (11.7)	879 (17.2)	902 (24.4)	546 (32.0)	1222 (46.6)	3582 (26.7)	142 (40.7)	3724 (27.1)
Less than 10th percentile at 36 wk	89 (31.7)	1825 (35.8)	1740 (47.0)	997 (58.5)	1796 (68.4)	6447 (48.1)	219 (62.8)	6666 (48.5)
Length								
*z* Score at birth, mean (SD)	0.1 (1.0)	0.1 (1.0)	−0.2 (1.1)	−0.3 (1.1)	−0.5 (1.2)	−0.2 (1.1)	−0.6 (1.3)	−0.2 (1.1)
*z* Score at 36 weeks, mean (SD)	−1.5 (1.1)	−1.5 (1.2)	−1.8 (1.1)	−2.1 (1.2)	−2.5 (1.3)	−1.8 (1.2)	−2.72 (1.5)	−1.9 (1.2)
*z* Score change from birth to 36 weeks less than −1.2	180 (66.2)	3264 (66.0)	2338 (66.4)	1101 (70.5)	1832 (77.2)	8715 (68.8)	252 (79.5)	8967 (69.0)
Less than 3rd percentile at 36 wk	96 (34.2)	1731 (34.3)	1638 (45.9)	887 (55.7)	1659 (68.7)	6011 (46.6)	235 (71.2)	6246 (47.2)
Less than 10th percentile at 36 wk	145 (51.6)	2763 (54.7)	2390 (66.9)	1206 (75.7)	2009 (83.2)	8513 (65.9)	273 (82.7)	8786 (66.3)
Head circumference								
*z* Score at birth, mean (SD)	0.2 (1.0)	0.0 (1.1)	−0.2 (1.1)	−0.3 (1.1)	−0.4 (1.1)	−0.2 (1.1)	−0.5 (1.2)	−0.2 (1.1)
*z* Score at 36 wk, mean (SD)	−1.1 (1.1)	−1.1 (1.2)	−1.3 (1.2)	−1.5 (1.2)	−2.0 (1.3)	−1.4 (1.2)	−2.2 (1.4)	−1.4 (1.2)
*z* Score change from birth to 36 wk <−1.2	139 (50.5)	2181 (43.6)	1600 (44.2)	828 (49.9)	1499 (59.7)	6247 (47.8)	223 (68.0)	6470 (48.3)
Less than percentile at 36 wk	56 (19.9)	1026 (20.2)	977 (26.7)	602 (35.8)	1297 (50.7)	3958 (29.9)	187 (55.5)	4145 (30.5)
Less than 10th percentile at 36 wk	114 (40.6)	2072 (40.9)	1797 (49.0)	1003 (59.6)	1817 (71.1)	6803 (51.3)	262 (77.7)	7065 (52.0)
**In-hospital morbidity^a^**								
Severity of critical illness at birth, mean (SD)^d^	0.635 (0.233)	0.533 (0.232)	0.379 (0.221)	0.300 (0.211)	0.224 (0.191)	0.404 (0.252)	0.186 (0.194)	0.389 (0.254)
Severity of critical illness change from birth to 7 DOLs, mean (SD)^d^	0.027 (0.133)	0.007 (0.132)	0.000 (0.138)	0.019 (0.132)	−0.022 (0.119)	−0.004 (0.132)	−0.033 (0.115)	−0.006 (0.131)
5-min Apgar score, median (IQR)	8 (7-8)	7 (6-8)	7 (6-8)	7 (5-8)	7 (5-8)	7 (6-8)	6 (4-8)	7 (6-8)
LOS	18 (6.2)	487 (9.0)	544 (14.0)	371 (20.7)	874 (32.4)	2294 (16.3)	442 (42.3)	2736 (18.1)
CLABSI^e^	4 (22.2)	66 (18.6)	83 (26.7)	49 (25.7)	140 (31.3)	342 (25.9)	109 (42.1)	451 (28.5)
IVH, grades 3-4	13 (4.5)	330 (6.1)	410 (10.7)	274 (15.4)	527 (19.6)	1554 (11.1)	336 (33.2)	1890 (12.6)
PVL	9 (3.1)	129 (2.4)	115 (3.0)	107 (6.0)	185 (6.9)	545 (3.9)	78 (7.7)	623 (4.1)
Physiologic BPD	112 (38.9)	1993 (37.9)	1943 (51.4)	1077 (62.1)	1916 (73.0)	7041 (51.4)	281 (80.1)	7322 (52.2)
BPD according to Jensen criteria								
None	155 (55.0)	2658 (52.8)	1321 (36.4)	446 (26.9)	444 (17.2)	5024 (38.1)	43 (13.1)	5067 (37.5)
Grade 1	75 (26.6)	1440 (28.6)	1239 (34.2)	594 (35.8)	842 (32.6)	4190 (31.8)	65 (19.8)	4255 (31.5)
Grade 2	44 (15.6)	784 (15.6)	845 (23.3)	468 (28.2)	938 (36.3)	3079 (23.3)	99 (30.2)	3178 (23.5)
Grade 3	8 (2.8)	151 (3.0)	220 (6.1)	153 (9.2)	362 (14.0)	894 (6.8)	121 (36.9)	1015 (7.5)
NEC stage 2 or higher^f^	16 (5.5)	318 (5.9)	253 (6.5)	166 (9.3)	419 (15.6)	1172 (8.3)	279 (26.7)	1451 (9.6)
SIP without NEC	1 (0.3)	6 (0.1)	14 (0.4)	45 (2.5)	316 (11.7)	382 (2.7)	231 (22.1)	613 (4.1)
Meningitis	0 (0.0)	41 (0.8)	27 (0.7)	20 (1.1)	46 (1.7)	134 (1.0)	20 (1.9)	154 (1.0)
Death	4 (1.4)	127 (2.4)	87 (2.2)	49 (2.7)	59 (2.2)	326 (2.3)	693 (66.3)	1019 (6.8)
Duration of hospital stay, median (IQR), d	56 (52-61)	59 (53-67)	65 (57-74)	70 (60-78)	75 (65-83)	64 (56-75)	21 (11-62)	64 (55-74)

Abbreviations: BPD, bronchopulmonary dysplasia; CLABSI, central line–associated bloodstream infection; DOL, day of life; IVH, intraventricular hemorrhage; LOS, late-onset sepsis; n, number in category; NA, not applicable; NEC, necrotizing enterocolitis; PMA, postmenstrual age; PVL, periventricular leukomalacia; SIP, spontaneous intestinal perforation.

^a^ Determined by 36 weeks’ PMA.

^b^ Collected for birth years 2012 to 2015.

^c^ The 36 weeks’ PMA follow-up growth measurements are restricted to 33 to 41 weeks’ PMA. Fenton growth curves were used for calculating relative growth.

^d^ Severity of critical illness is the predicted probability of survival to 36 weeks’ PMA without BPD based on neonatal characteristics collected on prespecified DOLs. Neonatal characteristics include gestational age, birth weight, sex, receipt of antenatal steroids, type of respiratory support, and fraction of inspired oxygen. See Greenberg et al^17^ for more information on the predicted probabilities of BPD grades and death at 36 weeks’ PMA.

^e^ CLABSI data collection began in 2016.

^f^ NEC determined according to the modified Bell staging criteria.

**Table 4.** Unadjusted and Adjusted Associations Between Full Enteral Feeding and Sepsis

Outcome	Association of time to achieve full enteral feeding (120 mL/kg/d) with in-hospital outcomes for neonates by 36 weeks’ PMA^a^		Association of number of days of full enteral feeding (120 mL/kg/d) in the first 28 d of life with in-hospital outcomes for neonates by 36 weeks’ PMA^b^	
	RR (95% CI)	ARR (95% CI)^c^	RR (95% CI)	ARR (95% CI)^c^
LOS	1.26 (1.24-1.28)	1.16 (1.14-1.18)	0.93 (0.92-0.93)	0.95 (0.95-0.96)
NEC stage 2 or higher^d^	1.23 (1.21-1.26)	1.20 (1.16-1.24)	0.94 (0.93-0.94)	0.94 (0.93-0.95)
Postnatal growth faltering^e^				
Weight	1.07 (1.06-1.08)	1.08 (1.07-1.09)	0.99 (0.98-0.99)	0.98 (0.98-0.98)
Length	1.04 (1.03-1.04)	1.03 (1.02-1.03)	0.99 (0.99-0.99)	0.99 (0.99-1.00)
Head circumference	1.07 (1.06-1.08)	1.07 (1.06-1.08)	0.98 (0.98-0.99)	0.98 (0.98-0.99)

Abbreviations: ARR, adjusted relative risk; LOS, late-onset sepsis; NEC, necrotizing enterocolitis; PMA, postmenstrual age; RR, relative risk (unadjusted).

^a^ Time to full enteral feeding is interpreted in increments of 7 days. An RR greater than 1.0 indicates an association in favor of more rapid attainment of full enteral feeds. For every 7-day delay in achieving full enteral feeding, the RRs of adverse clinical and growth outcomes increase.

^b^ An RR less than 1.0 indicates an association in favor of more rapid attainment of full enteral feeds. For every additional full enteral feeding day in the first 28 days after birth, the RRs of adverse clinical and growth outcomes decrease.

^c^ RRs from robust Poisson regression are adjusted for center as a fixed effect, birth year, severity of critical illness at birth, difference in severity of critical illness at birth and day of life 7, 5-minute Apgar score, small for gestational age, and maternal characteristics of educational level, insurance, hypertension, and insulin-treated diabetes. Severity of critical illness is the predicted probability of survival to 36 weeks’ PMA without bronchopulmonary dysplasia based on neonatal characteristics collected on prespecified days of life. Neonatal characteristics include gestational age, birth weight, sex, receipt of antenatal steroids, type of respiratory support, and fraction of inspired oxygen.

^d^ NEC determined according to the modified Bell staging criteria.

^e^ Postnatal growth faltering is defined as the change in Fenton growth curve *z* scores from birth to 36 weeks’ PMA follow-up of less than −1.2. The 36 weeks’ PMA follow-up growth measurements are restricted to data collected at 33 to 41 weeks’ PMA.

## Discussion

In this multicenter cohort of 11 349 EPT infants born before 28 completed weeks of gestation and 3753 very preterm infants born at 28 weeks of gestation, we found that shorter time to establish full enteral feeding was associated with a lower risk of LOS, NEC, and growth faltering in analyses adjusted for a proxy of illness severity and birth year. We also found that early full feeding was associated with a shorter duration of parenteral nutrition and a shorter length of hospital stay. In an exploratory analysis, we found that the practice of establishing full enteral feeding within the first 10 days after birth increased from 6.6% in 2012 to 26.3% in 2021, the absolute incidence of LOS decreased by 4.6 percentage points from 2012 to 2021, and the prevalence of growth faltering in head circumference is increasing over time.

Although we could not characterize the specific strategies applied in each NICU to favor the early establishment of full enteral feeding, these findings are broadly consistent with results from meta-analyses summarizing the effects of earlier^11^ and more rapid advancement of enteral feeding volumes.^12^ The meta-analysis on early progression of enteral feeding that included EPT infants along with very preterm infants (28-32 weeks of gestation) demonstrated that early achievement of full enteral feeding reduced the risk of severe infection (22% vs 31%; RR, 0.70; 95% CI, 0.56-0.87; n = 872; *P* = .001), the use of parenteral nutrition, and the length of hospital stay.^11^ However, unlike results from meta-analyses that did not show significant differences in the incidence of NEC between early and delayed feeding progression groups (8% vs 7%; RR, 1.17; 95% CI, 0.80-1.70)^11^ or between faster and slower progression of enteral feeding (5% vs 6%; RR, 1.06; 95% CI, 0.83-1.37),^12^ our analysis showed a potential risk reduction in NEC. Possible explanations include selection bias because our study population consisted of preterm infants treated in academic NICUs with likely greater illness complexity and longer times to achieve full enteral feeding compared with other cohorts.^18^ Consistent with this interpretation, an underpowered subgroup analysis^12^ of the Speed of Increasing Milk Feeds Trial (SIFT) suggested that high-risk preterm infants were less likely to develop NEC with faster feeding advancements (RR, 0.54; 95% CI, 0.24-1.24; *P* = .20), and a network meta-analysis^21^ of randomized clinical trials reported a lower risk of NEC with early initiation of enteral feeding (<72 hours after birth) and moderately early feeding advancements (72 hours to 7 days) using moderate advancement rates (20-29 mL/kg/d).

### Strengths and Limitations

This observational study with a large sample size possesses several strengths. A key strength is the consideration of a proxy of critical illness severity when evaluating the initiation and progression of enteral feeding.^1^ By adjusting for a proxy of critical illness during the first week after birth as a potential confounder, this analysis provides a more refined understanding of associations between early feeding and in-hospital outcomes in EPT infants. This approach is likely more appropriate than traditional regression models in which outcomes such as NEC or BPD, which may themselves be consequences of delayed feeding, are included as covariates. Additionally, the study moves beyond the conventional dichotomous classification of LOS based on the somewhat arbitrary 72-hour threshold, instead presenting LOS incidence by postnatal day. This approach provides greater granularity and reflects the complex nature of sepsis in preterm infants, a multifactorial disease often seen as unrelated to time to full enteral feeding. A further strength lies in the provision of a detailed breakdown of causative organisms and the inclusion of CONS cases, which constitute approximately half of LOS episodes. Recognizing the ongoing debate regarding whether CONS-associated LOS should be excluded due to its potential as a contaminant, this inclusive approach ensures a more accurate representation of infection burden. Differentiation between LOS and catheter-associated bloodstream infections in a subset of participants adds further depth to the analysis. The observed reduction in CONS-related LOS appears to coincide with an increased likelihood of sepsis caused by other organisms, particularly gram-negative pathogens. Additionally, the comprehensive reporting of postnatal growth faltering at 36 weeks’ PMA, using various definitions, provides critical information about growth trajectories during early life.^26^

Several limitations also warrant consideration. Adjusted analyses in observational studies cannot eliminate the possibility of reverse causation. The temporal sequence between NEC and LOS is unclear, although a lower risk of NEC might have been mediated by lower LOS incidence in infants achieving early full enteral feeding. Similarly, without knowing when the disease process begins, we could not determine whether early decreases in the probability of survival without BPD, our proxy of illness severity, indicated initial manifestations of adverse outcomes, such as LOS and NEC, or whether early increases in the probability of survival without BPD were the result of early full enteral feeding. Another limitation is that the covariate birth year may not fully account for evolving clinical practice. Accordingly, our illustrations of temporal changes should be considered exploratory because formal trend analyses were beyond the scope of this study.

Our operational definition of LOS also has limitations. Without documentation of specific clinical signs of infection,^22^ we were unable to determine whether the observed reduction in CONS sepsis in infants who achieved early full enteral feeding was primarily due to decreased exposure to potentially CONS-colonized indwelling catheters,^23,24^ improvements in blood culture collection technique resulting in less contamination during the study period, changes in antibiotic stewardship, or a true reduction in illness severity. When critical illness is less severe, clinicians may be more likely to classify a CONS-positive blood culture as a contaminant rather than true bacteremia. The observed prevalence of LOS is consistent with work from other high-income countries.^25^

Defining full enteral feeding as volume intakes of 120 mL/kg/d or greater represents an additional limitation, particularly because details about feeding type (eg, maternal, donor, and formula feeding), fortification, and administration (eg, timing over pump) were not reported. Using a somewhat arbitrary volume threshold without considering energy intake may not accurately reflect nutritional adequacy. With more NICUs transitioning from formula use to donor milk use to supplement maternal milk feeding, clinicians may be more comfortable initiating, advancing, and fortifying enteral feeds, potentially reducing the risk of postnatal growth faltering, but these data were not captured in our registry. This limitation highlights the need for future studies to identify specific components of early feeding, such as the ratio of maternal to donor milk and the timing of fortification, that may prevent substantial weight loss in the first 2 weeks after birth and boost linear and head growth throughout the NICU stay.^27^ To partially address the lack of information on feeding intolerance before or after the achievement of full enteral feeding, the study introduced the number of days receiving full enteral feeding within the first 28 days, offering a more comprehensive assessment of feeding tolerance across participants. Analyzing both time to full feeding and full enteral feeding days in relation to in-hospital outcomes enhances the reliability of our findings. Additionally, the absence of central line data in the registry, along with the exclusion of infants who were never fed (7% of the cohort), represent limitations that may introduce bias and affect the generalizability of our findings.

## Conclusions

Our study found that early full enteral feeding was associated with a lower risk of LOS and a reduced need for parenteral nutrition, all without increasing the risk of NEC. The available clinical evidence indicates that achieving full enteral feeding shortly after birth in EPT infants is feasible and beneficial. While weight trajectories have shown modest improvement with this practice, significant decreases in length and head circumference z scores from birth to 36 weeks’ PMA persist. Although causality cannot be implied in observational studies, findings such as these are critical for informing evidence-based practices and ensuring optimal nutritional strategies for EPT infants.

## Notes

Supplement 1. **eFigure. **Flow diagram **eTable. **Time to LOS episode by pathogen

Supplement 2. **Nonauthor Collaborators.** The Eunice Kennedy Shriver National Institute of Child Health and Human Development Neonatal Research Network members

Supplement 3.
Data Sharing Statement
