# Supplementary material for: Time to Full Enteral Feeds and Late-Onset Sepsis in Extremely Preterm Infants
Source: JAMA Netw Open. 2025 Nov 17;8(11):e2543940. doi: 10.1001/jamanetworkopen.2025.43940 (PMC12625685; doi:10.1001/jamanetworkopen.2025.43940)
Supplement: Supplement 1. — eFigure. Flow diagram eTable. Time to LOS episode by pathogen [file jamanetwopen-e2543940-s001.pdf]

Supplemental Online Content

Salas AA, Wiener LE, Trotta M, et al. Time to full enteral feeds and late-onset sepsis in extremely preterm infants. *JAMA Netw Open*. 2025;8(11):e2543940. doi:10.1001/jamanetworkopen.2025.43940

eFigure. Flow diagram

eTable. Time to LOS episode by pathogen

This supplemental material has been provided by the authors to give readers additional information about their work.

© 2025 Salas AA et al. *JAMA Network Open.*

eFigure. Flow diagram

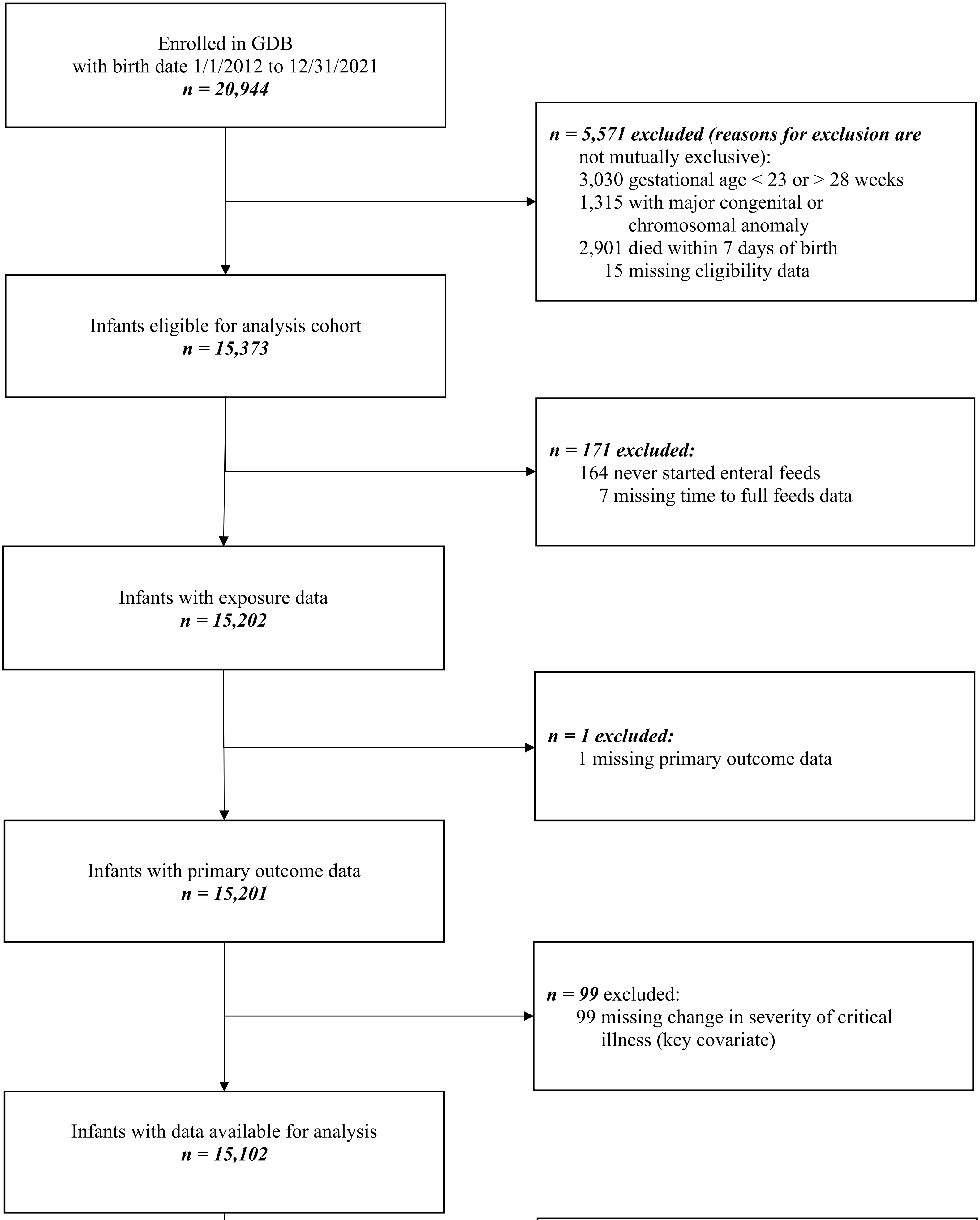

eTable. Time to LOS episode by pathogen

	Time in days from birth to first LOS N, median (IQR)										
**Birth year**	**CONS**	**MRSA/MSSA**	**E. coli**	**Klebsiella**	**Pseudomonas**	**Serratia**	**Enterobacter**	**Enterococcus**	**Candida**	**Strep B**	**Other**
**2012**	173, 14 (9, 27)	46, 15 (11, 20)	24, 9 (7.5, 14)	15, 13 (11, 16)	11, 19 (10, 24)	4, 18 (14, 24)	13, 20 (14, 32)	10, 23.5 (21, 28)	18, 16 (15, 21)	9, 48 (32, 56)	21, 26 (15, 38)
**2013**	129, 16 (11, 26)	45, 23 (13, 38)	19, 12 (6, 18)	9, 20 (16, 31)	9, 21 (10, 29)	6, 24 (18, 33)	7, 20 (14, 38)	5, 26 (21, 36)	8, 12 (9, 26)	8, 38 (12, 44)	13, 15 (9, 25)
**2014**	151, 16 (9, 28)	39, 25 (14, 42)	30, 9 (7, 22)	15, 10 (7, 36)	15, 18 (14, 24)	4, 22 (16, 28)	5, 17 (9, 19)	12, 14 (10, 28)	7, 11 (10, 16)	8, 37 (24, 42)	16, 16 (8, 29)
**2015**	139, 17 (10, 28)	56, 17 (12.5, 32.5)	30, 9.5 (7, 19)	18, 18 (12, 23)	6, 12 (10, 17)	7, 16 (12, 21)	9, 16 (8, 24)	8, 28 (10, 44)	12, 12 (8, 15)	7, 31 (14, 33)	15, 19 (12, 29)
**2016**	113, 17 (10, 29)	46, 17.5 (11, 33)	23, 12 (8, 38)	13, 15 (11, 21)	8, 11.5 (8, 21)	7, 15 (12, 25)	6, 34 (22, 46)	13, 26 (14, 47)	7, 7 (6, 19)	7, 30 (19, 55)	16, 22 (10, 39)
**2017**	112, 14 (9, 26)	47, 16 (12, 29)	29, 9 (8, 14)	12, 16 (12, 22)	3, 15 (10, 22)	6, 17 (11, 30)	4, 23 (12, 44)	10, 26 (21, 56)	14, 12 (8, 22)	8, 34 (24, 47)	15, 29 (9, 44)
**2018**	103, 13 (8, 21)	42, 17 (11, 27)	35, 10 (7, 18)	22, 14.5 (8, 20)	5, 9 (8, 16)	9, 18 (13, 32)	8, 22 (15, 28)	13, 28 (22, 46)	13, 10 (8, 13)	6, 40 (25, 54)	8, 14 (8, 23)
**2019**	105, 15 (9, 32)	44, 16.5 (11, 28.5)	35, 14 (8, 38)	22, 14 (10, 19)	12, 20 (14, 34)	7, 11 (9, 52)	7, 17 (15, 24)	7, 22 (13, 31)	11, 13 (8, 16)	9, 47 (27, 50)	13, 14 (6, 44)
**2020**	93, 19 (10, 28)	41, 18 (11, 32)	23, 10 (8, 13)	12, 18 (14, 30)	10, 16 (14, 22)	8, 14 (9, 36)	8, 17 (14, 22)	5, 47 (23, 68)	8, 11 (6, 16)	11, 30 (14, 38)	16, 16 (11, 32)
**2021**	98, 15 (10, 26)	42, 16.5 (11, 32)	26, 11.5 (8, 16)	16, 14 (8, 26)	6, 17 (11, 22)	5, 21 (15, 23)	4, 33 (19, 54)	13, 12 (10, 17)	5, 22 (8, 23)	6, 20 (15, 61)	14, 13 (10, 43)
**Overall**	1216, 15 (9, 27)	448, 18 (12, 32)	274, 10 (7, 19)	154, 15 (9, 22)	85, 17 (10, 23)	63, 16 (12, 25)	71, 19 (13, 31)	96, 22 (13, 39)	103, 12 (8, 18)	79, 33 (19, 51)	147, 18 (10, 38)

Abbreviations: LOS = late-onset sepsis; CONS = Coagulase-Negative Staphylococcus; MRSA = Methicillin-Resistant Staphylococcus aureus; MSSA = Methicillin-Sensitive Staphylococcus aureus

© 2025 Salas AA et al. *JAMA Network Open.*
