# Supplementary material for: Time to Full Enteral Feeds and Late-Onset Sepsis in Extremely Preterm Infants
Source: JAMA Netw Open. 2025 Nov 17;8(11):e2543940. doi: 10.1001/jamanetworkopen.2025.43940 (PMC12625685; doi:10.1001/jamanetworkopen.2025.43940)
Supplement: Supplement 2. — Nonauthor Collaborators. The Eunice Kennedy Shriver National Institute of Child Health and Human Development Neonatal Research Network members [file jamanetwopen-e2543940-s002.pdf]

Supplemental Online Content: Nonauthor Collaborators
*First name, last name, and suffix (if applicable) are required and will appear in PubMed.

*Group Name(s): The Eunice Kennedy Shriver National Institute of Child Health and Development Neonatal Research Network							
***First Name and Middle Initial(s)**	***Last Name**	***Suffix (eg, Jr, III)**	Academic Degrees	Institution	Location (city, state/province, country)	Role or Contribution, eg, chair, principal investigator	Group (if more than 1 Group listed in the byline) and/or Subgroup (eg, Steering Committee)
Richard A.	Polin		MD	Columbia University, College of Physicians and Surgeons, Division of Neonatology	New York, NY	Non-author contributor	
Abbot R.	Laptook		MD	Alpert Medical School of Brown University / Women & Infants Hospital of Rhode Island	Providence, RI	Non-author contributor	
Martin	Keszler		MD	Alpert Medical School of Brown University / Women & Infants Hospital of Rhode Island	Providence, RI	Non-author contributor	
Angelita M.	Hensman		PhD RNC-NIC	Alpert Medical School of Brown University / Women & Infants Hospital of Rhode Island	Providence, RI	Non-author contributor	
Elisa	Vieira		BSN RN	Alpert Medical School of Brown University / Women & Infants Hospital of Rhode Island	Providence, RI	Non-author contributor	
Lucille	St. Pierre		BS	Alpert Medical School of Brown University / Women & Infants Hospital of Rhode Island	Providence, RI	Non-author contributor	
Emilee	Little		RN	Alpert Medical School of Brown University / Women & Infants Hospital of Rhode Island	Providence, RI	Non-author contributor	

Supplemental Online Content: Nonauthor Collaborators
*First name, last name, and suffix (if applicable) are required and will appear in PubMed.

***First Name and Middle Initial(s)**	***Last Name**	***Suffix (eg, Jr, III)**	Academic Degrees	Institution	Location (city, state/province, country)	Role or Contribution, eg, chair, principal investigator	Group (if more than 1 Group listed in the byline) and/or Subgroup (eg, Steering Committee)
Anna Marie	Hibbs		MD	Case Western Reserve University / Rainbow Babies & Children's Hospital	Cleveland, OH	Non-author contributor	
Michele C.	Walsh		MD MS	Case Western Reserve University / Rainbow Babies & Children's Hospital	Cleveland, OH	Non-author contributor	
Deanne E.	Wilson-Costello		MD	Case Western Reserve University / Rainbow Babies & Children's Hospital	Cleveland, OH	Non-author contributor	
Nancy S.	Newman		RN	Case Western Reserve University / Rainbow Babies & Children's Hospital	Cleveland, OH	Non-author contributor	
Bonnie S.	Siner		RN	Case Western Reserve University / Rainbow Babies & Children's Hospital	Cleveland, OH	Non-author contributor	
Angelia	Williams			Case Western Reserve University / Rainbow Babies & Children's Hospital	Cleveland, OH	Non-author contributor	
William E.	Truog		MD	Children's Mercy Hospital / University of Missouri Kansas City School of Medicine / Kansas University Medical Center	Kansas City, MO	Non-author contributor	
Howard	Kilbride		MD	Children's Mercy Hospital / University of Missouri Kansas City School of Medicine / Kansas University Medical Center	Kansas City, MO	Non-author contributor	

*First name, last name, and suffix (if applicable) are required and will appear in PubMed.

***First Name and Middle Initial(s)**	***Last Name**	***Suffix (eg, Jr, III)**	Academic Degrees	Institution	Location (city, state/province, country)	Role or Contribution, eg, chair, principal investigator	Group (if more than 1 Group listed in the byline) and/or Subgroup (eg, Steering Committee)
Eugenia K.	Pallotto		MD MSCE	Children's Mercy Hospital / University of Missouri Kansas City School of Medicine / Kansas University Medical Center	Kansas City, MO	Non-author contributor	
Prabhu S.	Parimi		MD	Children's Mercy Hospital / University of Missouri Kansas City School of Medicine / Kansas University Medical Center	Kansas City, MO	Non-author contributor	
Cheri	Gauldin		RN BSN CCRC	Children's Mercy Hospital / University of Missouri Kansas City School of Medicine / Kansas University Medical Center	Kansas City, MO	Non-author contributor	
Lisa	Gaetano		RN MSN	Children's Mercy Hospital / University of Missouri Kansas City School of Medicine / Kansas University Medical Center	Kansas City, MO	Non-author contributor	
Anne	Holmes		RN MSN MBA-HCM CCRC	Children's Mercy Hospital / University of Missouri Kansas City School of Medicine / Kansas University Medical Center	Kansas City, MO	Non-author contributor	
Allison	Scott		BSN RNC-NIC	Children's Mercy Hospital / University of Missouri Kansas City School of Medicine / Kansas University Medical Center	Kansas City, MO	Non-author contributor	

*First name, last name, and suffix (if applicable) are required and will appear in PubMed.

***First Name and Middle Initial(s)**	***Last Name**	***Suffix (eg, Jr, III)**	Academic Degrees	Institution	Location (city, state/province, country)	Role or Contribution, eg, chair, principal investigator	Group (if more than 1 Group listed in the byline) and/or Subgroup (eg, Steering Committee)
Kurt	Schibler		MD	Cincinnati Children's Hospital Medical Center / University of Cincinnati Medical Center / Good Samaritan Hospital	Cincinnati, OH	Non-author contributor	
Stephanie L.	Merhar		MD MS	Cincinnati Children's Hospital Medical Center / University of Cincinnati Medical Center / Good Samaritan Hospital	Cincinnati, OH	Non-author contributor	
David	Russell		JD	Cincinnati Children's Hospital Medical Center / University of Cincinnati Medical Center / Good Samaritan Hospital	Cincinnati, OH	Non-author contributor	
Cathy	Grisby		BSN CCRC	Cincinnati Children's Hospital Medical Center / University of Cincinnati Medical Center / Good Samaritan Hospital	Cincinnati, OH	Non-author contributor	
Kristin	Kirker		CRC	Cincinnati Children's Hospital Medical Center / University of Cincinnati Medical Center / Good Samaritan Hospital	Cincinnati, OH	Non-author contributor	
Julia	Thompson		RN BSN	Cincinnati Children's Hospital Medical Center / University of Cincinnati Medical Center / Good Samaritan Hospital	Cincinnati, OH	Non-author contributor	
Traci	Beiersdorfer		RN BSN	Cincinnati Children's Hospital Medical Center / University of Cincinnati Medical Center / Good Samaritan Hospital	Cincinnati, OH	Non-author contributor	

Supplemental Online Content: Nonauthor Collaborators
*First name, last name, and suffix (if applicable) are required and will appear in PubMed.

***First Name and Middle Initial(s)**	***Last Name**	***Suffix (eg, Jr, III)**	Academic Degrees	Institution	Location (city, state/province, country)	Role or Contribution, eg, chair, principal investigator	Group (if more than 1 Group listed in the byline) and/or Subgroup (eg, Steering Committee)
Sandra	Wuertz		RN BSN	Cincinnati Children's Hospital Medical Center / University of Cincinnati Medical Center / Good Samaritan Hospital	Cincinnati, OH	Non-author contributor	
Juanita	Dudley		RN BSN	Cincinnati Children's Hospital Medical Center / University of Cincinnati Medical Center / Good Samaritan Hospital	Cincinnati, OH	Non-author contributor	
Lenora	Jackson		CRC	Cincinnati Children's Hospital Medical Center / University of Cincinnati Medical Center / Good Samaritan Hospital	Cincinnati, OH	Non-author contributor	
C. Michael	Cotten		MD MHS	Duke University School of Medicine / Duke University Hospital / University of North Carolina at Chapel Hill / WakeMed Health and Hospitals / Maynard Children's Hospital at East Carolina University Health	Durham, NC	Non-author contributor	
Ronald N.	Goldberg		MD	Duke University School of Medicine / Duke University Hospital / University of North Carolina at Chapel Hill / WakeMed Health and Hospitals / Maynard Children's Hospital at East Carolina University Health	Durham, NC	Non-author contributor	

Supplemental Online Content: Nonauthor Collaborators
*First name, last name, and suffix (if applicable) are required and will appear in PubMed.

***First Name and Middle Initial(s)**	***Last Name**	***Suffix (eg, Jr, III)**	Academic Degrees	Institution	Location (city, state/province, country)	Role or Contribution, eg, chair, principal investigator	Group (if more than 1 Group listed in the byline) and/or Subgroup (eg, Steering Committee)
Matthew M.	Laughon		MD MPH	Duke University School of Medicine / Duke University Hospital / University of North Carolina at Chapel Hill / WakeMed Health and Hospitals / Maynard Children's Hospital at East Carolina University Health	Durham, NC	Non-author contributor	
Joanne	Propst		RN JD	Duke University School of Medicine / Duke University Hospital / University of North Carolina at Chapel Hill / WakeMed Health and Hospitals / Maynard Children's Hospital at East Carolina University Health	Durham, NC	Non-author contributor	
Kimberley A.	Fisher		PhD FNP-BC IBCLC	Duke University School of Medicine / Duke University Hospital / University of North Carolina at Chapel Hill / WakeMed Health and Hospitals / Maynard Children's Hospital at East Carolina University Health	Durham, NC	Non-author contributor	

Supplemental Online Content: Nonauthor Collaborators
*First name, last name, and suffix (if applicable) are required and will appear in PubMed.

***First Name and Middle Initial(s)**	***Last Name**	***Suffix (eg, Jr, III)**	Academic Degrees	Institution	Location (city, state/province, country)	Role or Contribution, eg, chair, principal investigator	Group (if more than 1 Group listed in the byline) and/or Subgroup (eg, Steering Committee)
Melissa	Babilonia-Rosa		PhD	Duke University School of Medicine / Duke University Hospital / University of North Carolina at Chapel Hill / WakeMed Health and Hospitals / Maynard Children's Hospital at East Carolina University Health	Durham, NC	Non-author contributor	
Carl L.	Bose		MD	Duke University School of Medicine / Duke University Hospital / University of North Carolina at Chapel Hill / WakeMed Health and Hospitals / Maynard Children's Hospital at East Carolina University Health	Durham, NC	Non-author contributor	
Janice	Bernhardt		MS RN	Duke University School of Medicine / Duke University Hospital / University of North Carolina at Chapel Hill / WakeMed Health and Hospitals / Maynard Children's Hospital at East Carolina University Health	Durham, NC	Non-author contributor	

Supplemental Online Content: Nonauthor Collaborators
*First name, last name, and suffix (if applicable) are required and will appear in PubMed.

***First Name and Middle Initial(s)**	***Last Name**	***Suffix (eg, Jr, III)**	Academic Degrees	Institution	Location (city, state/province, country)	Role or Contribution, eg, chair, principal investigator	Group (if more than 1 Group listed in the byline) and/or Subgroup (eg, Steering Committee)
Gennie	Bose		RN	Duke University School of Medicine / Duke University Hospital / University of North Carolina at Chapel Hill / WakeMed Health and Hospitals / Maynard Children's Hospital at East Carolina University Health	Durham, NC	Non-author contributor	
Cindy	Clark		RN	Duke University School of Medicine / Duke University Hospital / University of North Carolina at Chapel Hill / WakeMed Health and Hospitals / Maynard Children's Hospital at East Carolina University Health	Durham, NC	Non-author contributor	
Jennifer	Talbert		MS RN BSN RDH	Duke University School of Medicine / Duke University Hospital / University of North Carolina at Chapel Hill / WakeMed Health and Hospitals / Maynard Children's Hospital at East Carolina University Health	Durham, NC	Non-author contributor	

*First name, last name, and suffix (if applicable) are required and will appear in PubMed.

***First Name and Middle Initial(s)**	***Last Name**	***Suffix (eg, Jr, III)**	Academic Degrees	Institution	Location (city, state/province, country)	Role or Contribution, eg, chair, principal investigator	Group (if more than 1 Group listed in the byline) and/or Subgroup (eg, Steering Committee)
Stephen D.	Kicklighter		MD	Duke University School of Medicine / Duke University Hospital / University of North Carolina at Chapel Hill / WakeMed Health and Hospitals / Maynard Children's Hospital at East Carolina University Health	Durham, NC	Non-author contributor	
Ginger	Rhodes-Ryan		ARNP MSN NNP-BC	Duke University School of Medicine / Duke University Hospital / University of North Carolina at Chapel Hill / WakeMed Health and Hospitals / Maynard Children's Hospital at East Carolina University Health	Durham, NC	Non-author contributor	
Donna	White		BSN RN-BC	Duke University School of Medicine / Duke University Hospital / University of North Carolina at Chapel Hill / WakeMed Health and Hospitals / Maynard Children's Hospital at East Carolina University Health	Durham, NC	Non-author contributor	

Supplemental Online Content: Nonauthor Collaborators
*First name, last name, and suffix (if applicable) are required and will appear in PubMed.

***First Name and Middle Initial(s)**	***Last Name**	***Suffix (eg, Jr, III)**	Academic Degrees	Institution	Location (city, state/province, country)	Role or Contribution, eg, chair, principal investigator	Group (if more than 1 Group listed in the byline) and/or Subgroup (eg, Steering Committee)
Ryan	Moore		MD	Duke University School of Medicine / Duke University Hospital / University of North Carolina at Chapel Hill / WakeMed Health and Hospitals / Maynard Children's Hospital at East Carolina University Health	Durham, NC	Non-author contributor	
Kelly	Bear		MD	Duke University School of Medicine / Duke University Hospital / University of North Carolina at Chapel Hill / WakeMed Health and Hospitals / Maynard Children's Hospital at East Carolina University Health	Durham, NC	Non-author contributor	
Sherry	Moseley		RN	Duke University School of Medicine / Duke University Hospital / University of North Carolina at Chapel Hill / WakeMed Health and Hospitals / Maynard Children's Hospital at East Carolina University Health	Durham, NC	Non-author contributor	

*First name, last name, and suffix (if applicable) are required and will appear in PubMed.

***First Name and Middle Initial(s)**	***Last Name**	***Suffix (eg, Jr, III)**	Academic Degrees	Institution	Location (city, state/province, country)	Role or Contribution, eg, chair, principal investigator	Group (if more than 1 Group listed in the byline) and/or Subgroup (eg, Steering Committee)
Vicki	Bergstedt		RN	Duke University School of Medicine / Duke University Hospital / University of North Carolina at Chapel Hill / WakeMed Health and Hospitals / Maynard Children's Hospital at East Carolina University Health	Durham, NC	Non-author contributor	
Joseph	Ginski		MD	Duke University School of Medicine / Duke University Hospital / University of North Carolina at Chapel Hill / WakeMed Health and Hospitals / Maynard Children's Hospital at East Carolina University Health	Durham, NC	Non-author contributor	
Weili	Chang		MD	Duke University School of Medicine / Duke University Hospital / University of North Carolina at Chapel Hill / WakeMed Health and Hospitals / Maynard Children's Hospital at East Carolina University Health	Durham, NC	Non-author contributor	

*First name, last name, and suffix (if applicable) are required and will appear in PubMed.

***First Name and Middle Initial(s)**	***Last Name**	***Suffix (eg, Jr, III)**	Academic Degrees	Institution	Location (city, state/province, country)	Role or Contribution, eg, chair, principal investigator	Group (if more than 1 Group listed in the byline) and/or Subgroup (eg, Steering Committee)
Juan	Guillen Hernandez		MD	Duke University School of Medicine / Duke University Hospital / University of North Carolina at Chapel Hill / WakeMed Health and Hospitals / Maynard Children's Hospital at East Carolina University Health	Durham, NC	Non-author contributor	
Ravi M.	Patel		MD MSc	Emory University / Children's Healthcare of Atlanta / Grady Memorial Hospital / Emory University Hospital Midtown	Atlanta, GA	Non-author contributor	
David P.	Carlton		MD	Emory University / Children's Healthcare of Atlanta / Grady Memorial Hospital / Emory University Hospital Midtown	Atlanta, GA	Non-author contributor	
Ellen C.	Hale		RN BS CCRC	Emory University / Children's Healthcare of Atlanta / Grady Memorial Hospital / Emory University Hospital Midtown	Atlanta, GA	Non-author contributor	
Yvonne	Loggins		RN	Emory University / Children's Healthcare of Atlanta / Grady Memorial Hospital / Emory University Hospital Midtown	Atlanta, GA	Non-author contributor	
Colleen	Mackie		BS RT	Emory University / Children's Healthcare of Atlanta / Grady Memorial Hospital / Emory University Hospital Midtown	Atlanta, GA	Non-author contributor	

Supplemental Online Content: Nonauthor Collaborators
*First name, last name, and suffix (if applicable) are required and will appear in PubMed.

***First Name and Middle Initial(s)**	***Last Name**	***Suffix (eg, Jr, III)**	Academic Degrees	Institution	Location (city, state/province, country)	Role or Contribution, eg, chair, principal investigator	Group (if more than 1 Group listed in the byline) and/or Subgroup (eg, Steering Committee)
Diane I.	Bottcher		RN MSN	Emory University / Children's Healthcare of Atlanta / Grady Memorial Hospital / Emory University Hospital Midtown	Atlanta, GA	Non-author contributor	
Andrew A.	Bremer		MD PhD	Eunice Kennedy Shriver National Institute of Child Health and Human Development	Bethesda, MD	Non-author contributor	
Rosemary D.	Higgins		MD	Eunice Kennedy Shriver National Institute of Child Health and Human Development	Bethesda, MD	Non-author contributor	
Stephanie Wilson	Archer		MA	Eunice Kennedy Shriver National Institute of Child Health and Human Development	Bethesda, MD	Non-author contributor	
Gregory M.	Sokol		MD	Indiana University / Methodist Hospital / Riley Hospital for Children at Indiana University Health / Eskenazi Health	Indianapolis, IN	Non-author contributor	
Dianne E.	Herron		RN CCRC	Indiana University / Methodist Hospital / Riley Hospital for Children at Indiana University Health / Eskenazi Health	Indianapolis, IN	Non-author contributor	
Susan	Gunn		NNP CCRC	Indiana University / Methodist Hospital / Riley Hospital for Children at Indiana University Health / Eskenazi Health	Indianapolis, IN	Non-author contributor	

Supplemental Online Content: Nonauthor Collaborators
*First name, last name, and suffix (if applicable) are required and will appear in PubMed.

***First Name and Middle Initial(s)**	***Last Name**	***Suffix (eg, Jr, III)**	Academic Degrees	Institution	Location (city, state/province, country)	Role or Contribution, eg, chair, principal investigator	Group (if more than 1 Group listed in the byline) and/or Subgroup (eg, Steering Committee)
Jeffery	Joyce		CCRC	Indiana University / Methodist Hospital / Riley Hospital for Children at Indiana University Health / Eskenazi Health	Indianapolis, IN	Non-author contributor	
Jon E.	Tyson		MD MPH	McGovern Medical School at The University of Texas Health Science Center at Houston / Children's Memorial Hermann Hospital / Memorial Hermann Southwest Hospital	Houston, TX	Non-author contributor	
Kathleen A.	Kennedy		MD MPH	McGovern Medical School at The University of Texas Health Science Center at Houston / Children's Memorial Hermann Hospital / Memorial Hermann Southwest Hospital	Houston, TX	Non-author contributor	
Amir M.	Khan		MD	McGovern Medical School at The University of Texas Health Science Center at Houston / Children's Memorial Hermann Hospital / Memorial Hermann Southwest Hospital	Houston, TX	Non-author contributor	

Supplemental Online Content: Nonauthor Collaborators
*First name, last name, and suffix (if applicable) are required and will appear in PubMed.

***First Name and Middle Initial(s)**	***Last Name**	***Suffix (eg, Jr, III)**	Academic Degrees	Institution	Location (city, state/province, country)	Role or Contribution, eg, chair, principal investigator	Group (if more than 1 Group listed in the byline) and/or Subgroup (eg, Steering Committee)
Elizabeth	Eason		MD	McGovern Medical School at The University of Texas Health Science Center at Houston / Children's Memorial Hermann Hospital / Memorial Hermann Southwest Hospital	Houston, TX	Non-author contributor	
Emily K.	Stephens		BSN RNC-NIC	McGovern Medical School at The University of Texas Health Science Center at Houston / Children's Memorial Hermann Hospital / Memorial Hermann Southwest Hospital	Houston, TX	Non-author contributor	
Georgia E.	McDavid		RN	McGovern Medical School at The University of Texas Health Science Center at Houston / Children's Memorial Hermann Hospital / Memorial Hermann Southwest Hospital	Houston, TX	Non-author contributor	
Donna	Hall		RN	McGovern Medical School at The University of Texas Health Science Center at Houston / Children's Memorial Hermann Hospital / Memorial Hermann Southwest Hospital	Houston, TX	Non-author contributor	

*First name, last name, and suffix (if applicable) are required and will appear in PubMed.

***First Name and Middle Initial(s)**	***Last Name**	***Suffix (eg, Jr, III)**	Academic Degrees	Institution	Location (city, state/province, country)	Role or Contribution, eg, chair, principal investigator	Group (if more than 1 Group listed in the byline) and/or Subgroup (eg, Steering Committee)
Apoorva	Mahatme		BS	McGovern Medical School at The University of Texas Health Science Center at Houston / Children's Memorial Hermann Hospital / Memorial Hermann Southwest Hospital	Houston, TX	Non-author contributor	
Karen	Martin		RN	McGovern Medical School at The University of Texas Health Science Center at Houston / Children's Memorial Hermann Hospital / Memorial Hermann Southwest Hospital	Houston, TX	Non-author contributor	
V Ilse	Reyna		IMG	McGovern Medical School at The University of Texas Health Science Center at Houston / Children's Memorial Hermann Hospital / Memorial Hermann Southwest Hospital	Houston, TX	Non-author contributor	
Debasree Sana	Boral		BS IMG	McGovern Medical School at The University of Texas Health Science Center at Houston / Children's Memorial Hermann Hospital / Memorial Hermann Southwest Hospital	Houston, TX	Non-author contributor	

*First name, last name, and suffix (if applicable) are required and will appear in PubMed.

***First Name and Middle Initial(s)**	***Last Name**	***Suffix (eg, Jr, III)**	Academic Degrees	Institution	Location (city, state/province, country)	Role or Contribution, eg, chair, principal investigator	Group (if more than 1 Group listed in the byline) and/or Subgroup (eg, Steering Committee)
Michelle	White		BSN RNC-NIC	McGovern Medical School at The University of Texas Health Science Center at Houston / Children's Memorial Hermann Hospital / Memorial Hermann Southwest Hospital	Houston, TX	Non-author contributor	
Sharon L.	Wright		MT	McGovern Medical School at The University of Texas Health Science Center at Houston / Children's Memorial Hermann Hospital / Memorial Hermann Southwest Hospital	Houston, TX	Non-author contributor	
Pablo J.	Sánchez		MD	Nationwide Children’s Hospital / Abigail Wexner Research Institute / Center for Perinatal Research / The Ohio State University College of Medicine / The Ohio State University Wexner Medical Center / Riverside Methodist Hospital	Columbus, OH	Non-author contributor	

Supplemental Online Content: Nonauthor Collaborators
*First name, last name, and suffix (if applicable) are required and will appear in PubMed.

***First Name and Middle Initial(s)**	***Last Name**	***Suffix (eg, Jr, III)**	Academic Degrees	Institution	Location (city, state/province, country)	Role or Contribution, eg, chair, principal investigator	Group (if more than 1 Group listed in the byline) and/or Subgroup (eg, Steering Committee)
Jonathan L.	Slaughter		MD MPH	Nationwide Children's Hospital / Abigail Wexner Research Institute / Center for Perinatal Research / The Ohio State University College of Medicine / The Ohio State University Wexner Medical Center / Riverside Methodist Hospital	Columbus, OH	Non-author contributor	
Leif D.	Nelin		MD	Nationwide Children's Hospital / Abigail Wexner Research Institute / Center for Perinatal Research / The Ohio State University College of Medicine / The Ohio State University Wexner Medical Center / Riverside Methodist Hospital	Columbus, OH	Non-author contributor	
Carl H.	Backes		MD	Nationwide Children's Hospital / Abigail Wexner Research Institute / Center for Perinatal Research / The Ohio State University College of Medicine / The Ohio State University Wexner Medical Center / Riverside Methodist Hospital	Columbus, OH	Non-author contributor	

*First name, last name, and suffix (if applicable) are required and will appear in PubMed.

***First Name and Middle Initial(s)**	***Last Name**	***Suffix (eg, Jr, III)**	Academic Degrees	Institution	Location (city, state/province, country)	Role or Contribution, eg, chair, principal investigator	Group (if more than 1 Group listed in the byline) and/or Subgroup (eg, Steering Committee)
Sudarshan R.	Jadcherla		MD	Nationwide Children's Hospital / Abigail Wexner Research Institute / Center for Perinatal Research / The Ohio State University College of Medicine / The Ohio State University Wexner Medical Center / Riverside Methodist Hospital	Columbus, OH	Non-author contributor	
Patricia	Luzader		RN	Nationwide Children's Hospital / Abigail Wexner Research Institute / Center for Perinatal Research / The Ohio State University College of Medicine / The Ohio State University Wexner Medical Center / Riverside Methodist Hospital	Columbus, OH	Non-author contributor	
Erna	Clark		BA	Nationwide Children's Hospital / Abigail Wexner Research Institute / Center for Perinatal Research / The Ohio State University College of Medicine / The Ohio State University Wexner Medical Center / Riverside Methodist Hospital	Columbus, OH	Non-author contributor	

*First name, last name, and suffix (if applicable) are required and will appear in PubMed.

***First Name and Middle Initial(s)**	***Last Name**	***Suffix (eg, Jr, III)**	Academic Degrees	Institution	Location (city, state/province, country)	Role or Contribution, eg, chair, principal investigator	Group (if more than 1 Group listed in the byline) and/or Subgroup (eg, Steering Committee)
Julie	Gutentag		RN	Nationwide Children's Hospital / Abigail Wexner Research Institute / Center for Perinatal Research / The Ohio State University College of Medicine / The Ohio State University Wexner Medical Center / Riverside Methodist Hospital	Columbus, OH	Non-author contributor	
Courtney	Park		RN	Nationwide Children's Hospital / Abigail Wexner Research Institute / Center for Perinatal Research / The Ohio State University College of Medicine / The Ohio State University Wexner Medical Center / Riverside Methodist Hospital	Columbus, OH	Non-author contributor	
Julie	Shadd		BA	Nationwide Children's Hospital / Abigail Wexner Research Institute / Center for Perinatal Research / The Ohio State University College of Medicine / The Ohio State University Wexner Medical Center / Riverside Methodist Hospital	Columbus, OH	Non-author contributor	

Supplemental Online Content: Nonauthor Collaborators
*First name, last name, and suffix (if applicable) are required and will appear in PubMed.

***First Name and Middle Initial(s)**	***Last Name**	***Suffix (eg, Jr, III)**	Academic Degrees	Institution	Location (city, state/province, country)	Role or Contribution, eg, chair, principal investigator	Group (if more than 1 Group listed in the byline) and/or Subgroup (eg, Steering Committee)
Melanie	Stein		BBA RRT	Nationwide Children's Hospital / Abigail Wexner Research Institute / Center for Perinatal Research / The Ohio State University College of Medicine / The Ohio State University Wexner Medical Center / Riverside Methodist Hospital	Columbus, OH	Non-author contributor	
Margaret	Sullivan		BA	Nationwide Children's Hospital / Abigail Wexner Research Institute / Center for Perinatal Research / The Ohio State University College of Medicine / The Ohio State University Wexner Medical Center / Riverside Methodist Hospital	Columbus, OH	Non-author contributor	
Rox Ann	Sullivan		RN BSN	Nationwide Children's Hospital / Abigail Wexner Research Institute / Center for Perinatal Research / The Ohio State University College of Medicine / The Ohio State University Wexner Medical Center / Riverside Methodist Hospital	Columbus, OH	Non-author contributor	

*First name, last name, and suffix (if applicable) are required and will appear in PubMed.

***First Name and Middle Initial(s)**	***Last Name**	***Suffix (eg, Jr, III)**	Academic Degrees	Institution	Location (city, state/province, country)	Role or Contribution, eg, chair, principal investigator	Group (if more than 1 Group listed in the byline) and/or Subgroup (eg, Steering Committee)
Laura	Marzec		MD	Nationwide Children's Hospital / Abigail Wexner Research Institute / Center for Perinatal Research / The Ohio State University College of Medicine / The Ohio State University Wexner Medical Center / Riverside Methodist Hospital	Columbus, OH	Non-author contributor	
Kyrstin	Warnimont		BS	Nationwide Children's Hospital / Abigail Wexner Research Institute / Center for Perinatal Research / The Ohio State University College of Medicine / The Ohio State University Wexner Medical Center / Riverside Methodist Hospital	Columbus, OH	Non-author contributor	
Demi R.	Beckford		MHS	Nationwide Children's Hospital / Abigail Wexner Research Institute / Center for Perinatal Research / The Ohio State University College of Medicine / The Ohio State University Wexner Medical Center / Riverside Methodist Hospital	Columbus, OH	Non-author contributor	

*First name, last name, and suffix (if applicable) are required and will appear in PubMed.

***First Name and Middle Initial(s)**	***Last Name**	***Suffix (eg, Jr, III)**	Academic Degrees	Institution	Location (city, state/province, country)	Role or Contribution, eg, chair, principal investigator	Group (if more than 1 Group listed in the byline) and/or Subgroup (eg, Steering Committee)
Hallie	Baugher		BS MSN	Nationwide Children's Hospital / Abigail Wexner Research Institute / Center for Perinatal Research / The Ohio State University College of Medicine / The Ohio State University Wexner Medical Center / Riverside Methodist Hospital	Columbus, OH	Non-author contributor	
Jessica	Purnell		BS CCRC	Nationwide Children's Hospital / Abigail Wexner Research Institute / Center for Perinatal Research / The Ohio State University College of Medicine / The Ohio State University Wexner Medical Center / Riverside Methodist Hospital	Columbus, OH	Non-author contributor	
Jennifer L.	Grothause		BA RN BSN	Nationwide Children's Hospital / Abigail Wexner Research Institute / Center for Perinatal Research / The Ohio State University College of Medicine / The Ohio State University Wexner Medical Center / Riverside Methodist Hospital	Columbus, OH	Non-author contributor	

*First name, last name, and suffix (if applicable) are required and will appear in PubMed.

***First Name and Middle Initial(s)**	***Last Name**	***Suffix (eg, Jr, III)**	Academic Degrees	Institution	Location (city, state/province, country)	Role or Contribution, eg, chair, principal investigator	Group (if more than 1 Group listed in the byline) and/or Subgroup (eg, Steering Committee)
Jacqueline	McCool			Nationwide Children's Hospital / Abigail Wexner Research Institute / Center for Perinatal Research / The Ohio State University College of Medicine / The Ohio State University Wexner Medical Center / Riverside Methodist Hospital	Columbus, OH	Non-author contributor	
Abhik	Das		PhD	RTI International	Research Triangle Park, NC	Non-author contributor	
Kristin M.	Zaterka-Baxter		RN BSN CCRP	RTI International	Research Triangle Park, NC	Non-author contributor	
Jenna	Gabrio		BS CCRP	RTI International	Research Triangle Park, NC	Non-author contributor	
David	Leblond		BS	RTI International	Research Triangle Park, NC	Non-author contributor	
Jeanette O'Donnell	Auman		BS	RTI International	Research Triangle Park, NC	Non-author contributor	
Amanda	Lewis			RTI International	Research Triangle Park, NC	Non-author contributor	
Valerie Y.	Chock		MD MS Epi	Stanford University / Lucile Packard Children's Hospital	Stanford, CA	Non-author contributor	
Krisa P.	Van Meurs		MD	Stanford University / Lucile Packard Children's Hospital	Stanford, CA	Non-author contributor	
David K.	Stevenson		MD	Stanford University / Lucile Packard Children's Hospital	Stanford, CA	Non-author contributor	
Alexis S.	Davis		MD MS Epi	Stanford University / Lucile Packard Children's Hospital	Stanford, CA	Non-author contributor	

Supplemental Online Content: Nonauthor Collaborators
*First name, last name, and suffix (if applicable) are required and will appear in PubMed.

***First Name and Middle Initial(s)**	***Last Name**	***Suffix (eg, Jr, III)**	Academic Degrees	Institution	Location (city, state/province, country)	Role or Contribution, eg, chair, principal investigator	Group (if more than 1 Group listed in the byline) and/or Subgroup (eg, Steering Committee)
Meera N.	Sankar		MD	Stanford University / Lucile Packard Children's Hospital	Stanford, CA	Non-author contributor	
M. Bethany	Ball		BS CCRC	Stanford University / Lucile Packard Children's Hospital	Stanford, CA	Non-author contributor	
Melinda S.	Proud		RCP	Stanford University / Lucile Packard Children's Hospital	Stanford, CA	Non-author contributor	
Elizabeth N.	Reichert		MA CCRC	Stanford University / Lucile Packard Children's Hospital	Stanford, CA	Non-author contributor	
Dharshi	Sivakumar		MD	Stanford University / Lucile Packard Children's Hospital	Stanford, CA	Non-author contributor	
R. Jordan	Williams		BA	Stanford University / Lucile Packard Children's Hospital	Stanford, CA	Non-author contributor	
Barbara P.	Recine		MA	Stanford University / Lucile Packard Children's Hospital	Stanford, CA	Non-author contributor	
Dona	Bahmani		CCRC	Stanford University / Lucile Packard Children's Hospital	Stanford, CA	Non-author contributor	
Jennifer E.	Chuck		MS	Stanford University / Lucile Packard Children's Hospital	Stanford, CA	Non-author contributor	
Lilia	Rutkowska		MA	Stanford University / Lucile Packard Children's Hospital	Stanford, CA	Non-author contributor	
Namasivayam	Ambalavanan		MD	University of Alabama at Birmingham Health System / Children’s Hospital of Alabama	Birmingham, AL	Non-author contributor	
Monica V.	Collins		RN BSN MaEd	University of Alabama at Birmingham Health System / Children’s Hospital of Alabama	Birmingham, AL	Non-author contributor	

*First name, last name, and suffix (if applicable) are required and will appear in PubMed.

***First Name and Middle Initial(s)**	***Last Name**	***Suffix (eg, Jr, III)**	Academic Degrees	Institution	Location (city, state/province, country)	Role or Contribution, eg, chair, principal investigator	Group (if more than 1 Group listed in the byline) and/or Subgroup (eg, Steering Committee)
Shirley S.	Cosby		RN BSN	University of Alabama at Birmingham Health System / Children's Hospital of Alabama	Birmingham, AL	Non-author contributor	
Cindie	Buie		RN BSN	University of Alabama at Birmingham Health System / Children's Hospital of Alabama	Birmingham, AL	Non-author contributor	
Sharon	Owen		RN ADN	University of Alabama at Birmingham Health System / Children's Hospital of Alabama	Birmingham, AL	Non-author contributor	
Tara	McNair		RN BSN	University of Alabama at Birmingham Health System / Children's Hospital of Alabama	Birmingham, AL	Non-author contributor	
Kelli P.	Hagood		RN BSN	University of Alabama at Birmingham Health System / Children's Hospital of Alabama	Birmingham, AL	Non-author contributor	
Uday	Devaskar		MD	University of California - Los Angeles / Mattel Children's Hospital / Santa Monica Hospital / Los Robles Hospital and Medical Center / Olive View Medical Center	Los Angeles, CA	Non-author contributor	

Supplemental Online Content: Nonauthor Collaborators
*First name, last name, and suffix (if applicable) are required and will appear in PubMed.

***First Name and Middle Initial(s)**	***Last Name**	***Suffix (eg, Jr, III)**	Academic Degrees	Institution	Location (city, state/province, country)	Role or Contribution, eg, chair, principal investigator	Group (if more than 1 Group listed in the byline) and/or Subgroup (eg, Steering Committee)
Meena	Garg		MD	University of California - Los Angeles / Mattel Children's Hospital / Santa Monica Hospital / Los Robles Hospital and Medical Center / Olive View Medical Center	Los Angeles, CA	Non-author contributor	
Teresa	Chanlaw		MPH	University of California - Los Angeles / Mattel Children's Hospital / Santa Monica Hospital / Los Robles Hospital and Medical Center / Olive View Medical Center	Los Angeles, CA	Non-author contributor	
Rachel	Geller		RN BSN	University of California - Los Angeles / Mattel Children's Hospital / Santa Monica Hospital / Los Robles Hospital and Medical Center / Olive View Medical Center	Los Angeles, CA	Non-author contributor	
Edward F.	Bell		MD	University of Iowa / Sanford Health	Iowa City, IA	Non-author contributor	
Tarah T.	Colaizy		MD MPH	University of Iowa / Sanford Health	Iowa City, IA	Non-author contributor	
Patrick J.	McNamara		MB BCH BAO DCH MSc (Paeds) MRCP MRCPCH	University of Iowa / Sanford Health	Iowa City, IA	Non-author contributor	

Supplemental Online Content: Nonauthor Collaborators
*First name, last name, and suffix (if applicable) are required and will appear in PubMed.

***First Name and Middle Initial(s)**	***Last Name**	***Suffix (eg, Jr, III)**	Academic Degrees	Institution	Location (city, state/province, country)	Role or Contribution, eg, chair, principal investigator	Group (if more than 1 Group listed in the byline) and/or Subgroup (eg, Steering Committee)
Karen J.	Johnson		RN BSN	University of Iowa / Sanford Health	Iowa City, IA	Non-author contributor	
Mendi L.	Schmelzel		MSN RN	University of Iowa / Sanford Health	Iowa City, IA	Non-author contributor	
Jacky R.	Walker		RN	University of Iowa / Sanford Health	Iowa City, IA	Non-author contributor	
Claire A.	Goeke		DNP ARNP	University of Iowa / Sanford Health	Iowa City, IA	Non-author contributor	
Sarah E.	Faruqui		MSN RN	University of Iowa / Sanford Health	Iowa City, IA	Non-author contributor	
Brenda J.	Coulter		RN	University of Iowa / Sanford Health	Iowa City, IA	Non-author contributor	
Michelle L.	Baack		MD	University of Iowa / Sanford Health	Iowa City, IA	Non-author contributor	
Chelsey	Elenkiwich		NNP APRN CNP	University of Iowa / Sanford Health	Iowa City, IA	Non-author contributor	
Megan M.	Henning		RN BSN	University of Iowa / Sanford Health	Iowa City, IA	Non-author contributor	
Megan	Broadbent		RN BSN	University of Iowa / Sanford Health	Iowa City, IA	Non-author contributor	
Sarah	Van Muyden		RN BSN	University of Iowa / Sanford Health	Iowa City, IA	Non-author contributor	
Kristi L.	Watterberg		MD	University of New Mexico Health Sciences Center	Albuquerque, NM	Non-author contributor	
Janell	Fuller		MD	University of New Mexico Health Sciences Center	Albuquerque, NM	Non-author contributor	
Robin K.	Ohls		MD	University of New Mexico Health Sciences Center	Albuquerque, NM	Non-author contributor	

Supplemental Online Content: Nonauthor Collaborators
*First name, last name, and suffix (if applicable) are required and will appear in PubMed.

***First Name and Middle Initial(s)**	***Last Name**	***Suffix (eg, Jr, III)**	Academic Degrees	Institution	Location (city, state/province, country)	Role or Contribution, eg, chair, principal investigator	Group (if more than 1 Group listed in the byline) and/or Subgroup (eg, Steering Committee)
Sandra Sundquist	Beauman		MSN RNC-NIC	University of New Mexico Health Sciences Center	Albuquerque, NM	Non-author contributor	
Conra	Backstrom Lacy		RN	University of New Mexico Health Sciences Center	Albuquerque, NM	Non-author contributor	
Mary	Hanson		RN BSN	University of New Mexico Health Sciences Center	Albuquerque, NM	Non-author contributor	
Elizabeth	Kuan		RN BSN	University of New Mexico Health Sciences Center	Albuquerque, NM	Non-author contributor	
Sara B.	DeMauro		MD MSCE	University of Pennsylvania / Hospital of the University of Pennsylvania / Pennsylvania Hospital / Children’s Hospital of Philadelphia / Virtua Voorhees Hospital	Philadelphia, PA	Non-author contributor	
Eric C.	Eichenwald		MD	University of Pennsylvania / Hospital of the University of Pennsylvania / Pennsylvania Hospital / Children’s Hospital of Philadelphia / Virtua Voorhees Hospital	Philadelphia, PA	Non-author contributor	
Barbara	Schmidt		MD MSc	University of Pennsylvania / Hospital of the University of Pennsylvania / Pennsylvania Hospital / Children’s Hospital of Philadelphia / Virtua Voorhees Hospital	Philadelphia, PA	Non-author contributor	

*First name, last name, and suffix (if applicable) are required and will appear in PubMed.

***First Name and Middle Initial(s)**	***Last Name**	***Suffix (eg, Jr, III)**	Academic Degrees	Institution	Location (city, state/province, country)	Role or Contribution, eg, chair, principal investigator	Group (if more than 1 Group listed in the byline) and/or Subgroup (eg, Steering Committee)
Haresh	Kirpalani		MB MSc	University of Pennsylvania / Hospital of the University of Pennsylvania / Pennsylvania Hospital / Children's Hospital of Philadelphia / Virtua Voorhees Hospital	Philadelphia, PA	Non-author contributor	
Soraya	Abbasi		MD	University of Pennsylvania / Hospital of the University of Pennsylvania / Pennsylvania Hospital / Children's Hospital of Philadelphia / Virtua Voorhees Hospital	Philadelphia, PA	Non-author contributor	
Christine	Catts		CRNP	University of Pennsylvania / Hospital of the University of Pennsylvania / Pennsylvania Hospital / Children's Hospital of Philadelphia / Virtua Voorhees Hospital	Philadelphia, PA	Non-author contributor	
Aasma S.	Chaudhary		BS RRT	University of Pennsylvania / Hospital of the University of Pennsylvania / Pennsylvania Hospital / Children's Hospital of Philadelphia / Virtua Voorhees Hospital	Philadelphia, PA	Non-author contributor	
Megan A.	Dhawan		MSN CRNP	University of Pennsylvania / Hospital of the University of Pennsylvania / Pennsylvania Hospital / Children's Hospital of Philadelphia / Virtua Voorhees Hospital	Philadelphia, PA	Non-author contributor	

*First name, last name, and suffix (if applicable) are required and will appear in PubMed.

***First Name and Middle Initial(s)**	***Last Name**	***Suffix (eg, Jr, III)**	Academic Degrees	Institution	Location (city, state/province, country)	Role or Contribution, eg, chair, principal investigator	Group (if more than 1 Group listed in the byline) and/or Subgroup (eg, Steering Committee)
Sarvin	Ghavam		MD	University of Pennsylvania / Hospital of the University of Pennsylvania / Pennsylvania Hospital / Children's Hospital of Philadelphia / Virtua Voorhees Hospital	Philadelphia, PA	Non-author contributor	
Antoinette	Mancini		RN BSN CCRC	University of Pennsylvania / Hospital of the University of Pennsylvania / Pennsylvania Hospital / Children's Hospital of Philadelphia / Virtua Voorhees Hospital	Philadelphia, PA	Non-author contributor	
Jonathan	Snyder		RN BSN	University of Pennsylvania / Hospital of the University of Pennsylvania / Pennsylvania Hospital / Children's Hospital of Philadelphia / Virtua Voorhees Hospital	Philadelphia, PA	Non-author contributor	
Carl T.	D'Angio		MD	University of Rochester Medical Center / Golisano Children's Hospital / University at Buffalo John R. Oishei Children's Hospital	Rochester, NY / Buffalo, NY	Non-author contributor	
Ronnie	Guillet		MD PhD	University of Rochester Medical Center / Golisano Children's Hospital / University at Buffalo John R. Oishei Children's Hospital	Rochester, NY / Buffalo, NY	Non-author contributor	

Supplemental Online Content: Nonauthor Collaborators
*First name, last name, and suffix (if applicable) are required and will appear in PubMed.

***First Name and Middle Initial(s)**	***Last Name**	***Suffix (eg, Jr, III)**	Academic Degrees	Institution	Location (city, state/province, country)	Role or Contribution, eg, chair, principal investigator	Group (if more than 1 Group listed in the byline) and/or Subgroup (eg, Steering Committee)
Anne Marie	Reynolds		MD MPH	University of Rochester Medical Center / Golisano Children's Hospital / University at Buffalo John R. Oishei Children's Hospital	Rochester, NY / Buffalo, NY	Non-author contributor	
Satyan	Lakshminrusimha		MD	University of Rochester Medical Center / Golisano Children's Hospital / University at Buffalo John R. Oishei Children's Hospital	Rochester, NY / Buffalo, NY	Non-author contributor	
Holly I.M.	Wadkins		MA	University of Rochester Medical Center / Golisano Children's Hospital / University at Buffalo John R. Oishei Children's Hospital	Rochester, NY / Buffalo, NY	Non-author contributor	
Michael G.	Sacilowski		MAT CCRC	University of Rochester Medical Center / Golisano Children's Hospital / University at Buffalo John R. Oishei Children's Hospital	Rochester, NY / Buffalo, NY	Non-author contributor	
Mary	Rowan		RN	University of Rochester Medical Center / Golisano Children's Hospital / University at Buffalo John R. Oishei Children's Hospital	Rochester, NY / Buffalo, NY	Non-author contributor	
Rosemary	Jensen			University of Rochester Medical Center / Golisano Children's Hospital / University at Buffalo John R. Oishei Children's Hospital	Rochester, NY / Buffalo, NY	Non-author contributor	

*First name, last name, and suffix (if applicable) are required and will appear in PubMed.

***First Name and Middle Initial(s)**	***Last Name**	***Suffix (eg, Jr, III)**	Academic Degrees	Institution	Location (city, state/province, country)	Role or Contribution, eg, chair, principal investigator	Group (if more than 1 Group listed in the byline) and/or Subgroup (eg, Steering Committee)
Diane	Prinzing		AAS	University of Rochester Medical Center / Golisano Children's Hospital / University at Buffalo John R. Oishei Children's Hospital	Rochester, NY / Buffalo, NY	Non-author contributor	
Ann Marie	Scorsone		MS CCRC	University of Rochester Medical Center / Golisano Children's Hospital / University at Buffalo John R. Oishei Children's Hospital	Rochester, NY / Buffalo, NY	Non-author contributor	
Kyle	Binion		BS	University of Rochester Medical Center / Golisano Children's Hospital / University at Buffalo John R. Oishei Children's Hospital	Rochester, NY / Buffalo, NY	Non-author contributor	
Stephanie	Guilford		BS	University of Rochester Medical Center / Golisano Children's Hospital / University at Buffalo John R. Oishei Children's Hospital	Rochester, NY / Buffalo, NY	Non-author contributor	
Constance	Orme			University of Rochester Medical Center / Golisano Children's Hospital / University at Buffalo John R. Oishei Children's Hospital	Rochester, NY / Buffalo, NY	Non-author contributor	
Premini	Sabaratnam		MPH	University of Rochester Medical Center / Golisano Children's Hospital / University at Buffalo John R. Oishei Children's Hospital	Rochester, NY / Buffalo, NY	Non-author contributor	

Supplemental Online Content: Nonauthor Collaborators
*First name, last name, and suffix (if applicable) are required and will appear in PubMed.

***First Name and Middle Initial(s)**	***Last Name**	***Suffix (eg, Jr, III)**	Academic Degrees	Institution	Location (city, state/province, country)	Role or Contribution, eg, chair, principal investigator	Group (if more than 1 Group listed in the byline) and/or Subgroup (eg, Steering Committee)
Daisy	Rochez		BS MHA	University of Rochester Medical Center / Golisano Children's Hospital / University at Buffalo John R. Oishei Children's Hospital	Rochester, NY / Buffalo, NY	Non-author contributor	
Alison	Kent		BMBS FRACP MD	University of Rochester Medical Center / Golisano Children's Hospital / University at Buffalo John R. Oishei Children's Hospital	Rochester, NY / Buffalo, NY	Non-author contributor	
Rachel	Jones			University of Rochester Medical Center / Golisano Children's Hospital / University at Buffalo John R. Oishei Children's Hospital	Rochester, NY / Buffalo, NY	Non-author contributor	
Elizabeth	Boylin		BA	University of Rochester Medical Center / Golisano Children's Hospital / University at Buffalo John R. Oishei Children's Hospital	Rochester, NY / Buffalo, NY	Non-author contributor	
Jennifer	Kachelmeyer		BS	University of Rochester Medical Center / Golisano Children's Hospital / University at Buffalo John R. Oishei Children's Hospital	Rochester, NY / Buffalo, NY	Non-author contributor	
Emily	Li		BA	University of Rochester Medical Center / Golisano Children's Hospital / University at Buffalo John R. Oishei Children's Hospital	Rochester, NY / Buffalo, NY	Non-author contributor	

*First name, last name, and suffix (if applicable) are required and will appear in PubMed.

***First Name and Middle Initial(s)**	***Last Name**	***Suffix (eg, Jr, III)**	Academic Degrees	Institution	Location (city, state/province, country)	Role or Contribution, eg, chair, principal investigator	Group (if more than 1 Group listed in the byline) and/or Subgroup (eg, Steering Committee)
Julieanne	Hunn		BS	University of Rochester Medical Center / Golisano Children's Hospital / University at Buffalo John R. Oishei Children's Hospital	Rochester, NY / Buffalo, NY	Non-author contributor	
Melissa	Bowman		RN	University of Rochester Medical Center / Golisano Children's Hospital / University at Buffalo John R. Oishei Children's Hospital	Rochester, NY / Buffalo, NY	Non-author contributor	
Ashley	Williams		MS Ed	University of Rochester Medical Center / Golisano Children's Hospital / University at Buffalo John R. Oishei Children's Hospital	Rochester, NY / Buffalo, NY	Non-author contributor	
Kelsey	Voelker		MPH	University of Rochester Medical Center / Golisano Children's Hospital / University at Buffalo John R. Oishei Children's Hospital	Rochester, NY / Buffalo, NY	Non-author contributor	
Deanna	Maffett		RN	University of Rochester Medical Center / Golisano Children's Hospital / University at Buffalo John R. Oishei Children's Hospital	Rochester, NY / Buffalo, NY	Non-author contributor	
Myra H.	Wyckoff		MD	University of Texas Southwestern Medical Center / Parkland Health & Hospital System / Children's Medical Center Dallas	Dallas, TX	Non-author contributor	

*First name, last name, and suffix (if applicable) are required and will appear in PubMed.

***First Name and Middle Initial(s)**	***Last Name**	***Suffix (eg, Jr, III)**	Academic Degrees	Institution	Location (city, state/province, country)	Role or Contribution, eg, chair, principal investigator	Group (if more than 1 Group listed in the byline) and/or Subgroup (eg, Steering Committee)
Luc P.	Brion		MD	University of Texas Southwestern Medical Center / Parkland Health & Hospital System / Children's Medical Center Dallas	Dallas, TX	Non-author contributor	
Lijun	Chen		RN PhD	University of Texas Southwestern Medical Center / Parkland Health & Hospital System / Children's Medical Center Dallas	Dallas, TX	Non-author contributor	
Maria M.	DeLeon		RN BSN	University of Texas Southwestern Medical Center / Parkland Health & Hospital System / Children's Medical Center Dallas	Dallas, TX	Non-author contributor	
Joann	Duran		MSN RN BSN	University of Texas Southwestern Medical Center / Parkland Health & Hospital System / Children's Medical Center Dallas	Dallas, TX	Non-author contributor	
Frances	Eubanks		RN BSN	University of Texas Southwestern Medical Center / Parkland Health & Hospital System / Children's Medical Center Dallas	Dallas, TX	Non-author contributor	
Michelle	Harrod		MSN RN BSN	University of Texas Southwestern Medical Center / Parkland Health & Hospital System / Children's Medical Center Dallas	Dallas, TX	Non-author contributor	

Supplemental Online Content: Nonauthor Collaborators
*First name, last name, and suffix (if applicable) are required and will appear in PubMed.

***First Name and Middle Initial(s)**	***Last Name**	***Suffix (eg, Jr, III)**	Academic Degrees	Institution	Location (city, state/province, country)	Role or Contribution, eg, chair, principal investigator	Group (if more than 1 Group listed in the byline) and/or Subgroup (eg, Steering Committee)
Lara	Pavageau		MD	University of Texas Southwestern Medical Center / Parkland Health & Hospital System / Children's Medical Center Dallas	Dallas, TX	Non-author contributor	
Pollieanna	Sepulveda		RN BSN	University of Texas Southwestern Medical Center / Parkland Health & Hospital System / Children's Medical Center Dallas	Dallas, TX	Non-author contributor	
Diana M.	Vasil		MSN RNC-NIC BSN	University of Texas Southwestern Medical Center / Parkland Health & Hospital System / Children's Medical Center Dallas	Dallas, TX	Non-author contributor	
Robin K.	Ohls		MD	University of Utah Medical Center / Intermountain Medical Center / McKay-Dee Hospital / Utah Valley Hospital / Primary Children's Medical Center	Salt Lake City, UT	Non-author contributor	
Erick B.	Gerday		MD	University of Utah Medical Center / Intermountain Medical Center / McKay-Dee Hospital / Utah Valley Hospital / Primary Children's Medical Center	Salt Lake City, UT	Non-author contributor	

*First name, last name, and suffix (if applicable) are required and will appear in PubMed.

***First Name and Middle Initial(s)**	***Last Name**	***Suffix (eg, Jr, III)**	Academic Degrees	Institution	Location (city, state/province, country)	Role or Contribution, eg, chair, principal investigator	Group (if more than 1 Group listed in the byline) and/or Subgroup (eg, Steering Committee)
Bradley A.	Yoder		MD	University of Utah Medical Center / Intermountain Medical Center / McKay-Dee Hospital / Utah Valley Hospital / Primary Children's Medical Center	Salt Lake City, UT	Non-author contributor	
Mariana	Baserga		MD MSCI	University of Utah Medical Center / Intermountain Medical Center / McKay-Dee Hospital / Utah Valley Hospital / Primary Children's Medical Center	Salt Lake City, UT	Non-author contributor	
Stephen D.	Minton		MD	University of Utah Medical Center / Intermountain Medical Center / McKay-Dee Hospital / Utah Valley Hospital / Primary Children's Medical Center	Salt Lake City, UT	Non-author contributor	
Mark J.	Sheffield		MD	University of Utah Medical Center / Intermountain Medical Center / McKay-Dee Hospital / Utah Valley Hospital / Primary Children's Medical Center	Salt Lake City, UT	Non-author contributor	
Carrie A.	Rau		RN BSN CCRC	University of Utah Medical Center / Intermountain Medical Center / McKay-Dee Hospital / Utah Valley Hospital / Primary Children's Medical Center	Salt Lake City, UT	Non-author contributor	

*First name, last name, and suffix (if applicable) are required and will appear in PubMed.

***First Name and Middle Initial(s)**	***Last Name**	***Suffix (eg, Jr, III)**	Academic Degrees	Institution	Location (city, state/province, country)	Role or Contribution, eg, chair, principal investigator	Group (if more than 1 Group listed in the byline) and/or Subgroup (eg, Steering Committee)
Jill	Burnett		RNC BSN	University of Utah Medical Center / Intermountain Medical Center / McKay-Dee Hospital / Utah Valley Hospital / Primary Children's Medical Center	Salt Lake City, UT	Non-author contributor	
Susan	Christensen		RN	University of Utah Medical Center / Intermountain Medical Center / McKay-Dee Hospital / Utah Valley Hospital / Primary Children's Medical Center	Salt Lake City, UT	Non-author contributor	
Kathleen	Coleman		RN	University of Utah Medical Center / Intermountain Medical Center / McKay-Dee Hospital / Utah Valley Hospital / Primary Children's Medical Center	Salt Lake City, UT	Non-author contributor	
Brandy	Davis		RN BSN	University of Utah Medical Center / Intermountain Medical Center / McKay-Dee Hospital / Utah Valley Hospital / Primary Children's Medical Center	Salt Lake City, UT	Non-author contributor	
Jennifer O.	Elmont		RN BSN	University of Utah Medical Center / Intermountain Medical Center / McKay-Dee Hospital / Utah Valley Hospital / Primary Children's Medical Center	Salt Lake City, UT	Non-author contributor	

*First name, last name, and suffix (if applicable) are required and will appear in PubMed.

***First Name and Middle Initial(s)**	***Last Name**	***Suffix (eg, Jr, III)**	Academic Degrees	Institution	Location (city, state/province, country)	Role or Contribution, eg, chair, principal investigator	Group (if more than 1 Group listed in the byline) and/or Subgroup (eg, Steering Committee)
Barbara L.	Francom		RN BSN	University of Utah Medical Center / Intermountain Medical Center / McKay-Dee Hospital / Utah Valley Hospital / Primary Children's Medical Center	Salt Lake City, UT	Non-author contributor	
Jamie	Jordan		RN BSN	University of Utah Medical Center / Intermountain Medical Center / McKay-Dee Hospital / Utah Valley Hospital / Primary Children's Medical Center	Salt Lake City, UT	Non-author contributor	
Manndi C.	Loertscher		BS CCRP	University of Utah Medical Center / Intermountain Medical Center / McKay-Dee Hospital / Utah Valley Hospital / Primary Children's Medical Center	Salt Lake City, UT	Non-author contributor	
Trisha	Marchant		RNC BSN	University of Utah Medical Center / Intermountain Medical Center / McKay-Dee Hospital / Utah Valley Hospital / Primary Children's Medical Center	Salt Lake City, UT	Non-author contributor	
Earl	Maxson		RN CCRN	University of Utah Medical Center / Intermountain Medical Center / McKay-Dee Hospital / Utah Valley Hospital / Primary Children's Medical Center	Salt Lake City, UT	Non-author contributor	

*First name, last name, and suffix (if applicable) are required and will appear in PubMed.

***First Name and Middle Initial(s)**	***Last Name**	***Suffix (eg, Jr, III)**	Academic Degrees	Institution	Location (city, state/province, country)	Role or Contribution, eg, chair, principal investigator	Group (if more than 1 Group listed in the byline) and/or Subgroup (eg, Steering Committee)
Kandace M.	McGrath		BS	University of Utah Medical Center / Intermountain Medical Center / McKay-Dee Hospital / Utah Valley Hospital / Primary Children's Medical Center	Salt Lake City, UT	Non-author contributor	
Hena G.	Mickelsen		BA	University of Utah Medical Center / Intermountain Medical Center / McKay-Dee Hospital / Utah Valley Hospital / Primary Children's Medical Center	Salt Lake City, UT	Non-author contributor	
D. Melody	Parry		RN BSN	University of Utah Medical Center / Intermountain Medical Center / McKay-Dee Hospital / Utah Valley Hospital / Primary Children's Medical Center	Salt Lake City, UT	Non-author contributor	
Susan T.	Schaefer		RN BSN RRT	University of Utah Medical Center / Intermountain Medical Center / McKay-Dee Hospital / Utah Valley Hospital / Primary Children's Medical Center	Salt Lake City, UT	Non-author contributor	
Katherine	Tice		RN BSN	University of Utah Medical Center / Intermountain Medical Center / McKay-Dee Hospital / Utah Valley Hospital / Primary Children's Medical Center	Salt Lake City, UT	Non-author contributor	

*First name, last name, and suffix (if applicable) are required and will appear in PubMed.

***First Name and Middle Initial(s)**	***Last Name**	***Suffix (eg, Jr, III)**	Academic Degrees	Institution	Location (city, state/province, country)	Role or Contribution, eg, chair, principal investigator	Group (if more than 1 Group listed in the byline) and/or Subgroup (eg, Steering Committee)
Kimberlee	Weaver-Lewis		RN MS	University of Utah Medical Center / Intermountain Medical Center / McKay-Dee Hospital / Utah Valley Hospital / Primary Children’s Medical Center	Salt Lake City, UT	Non-author contributor	
Kathryn D.	Woodbury		RN BSN	University of Utah Medical Center / Intermountain Medical Center / McKay-Dee Hospital / Utah Valley Hospital / Primary Children’s Medical Center	Salt Lake City, UT	Non-author contributor	
Lisa M.	Bell		RN BSN	University of Utah Medical Center / Intermountain Medical Center / McKay-Dee Hospital / Utah Valley Hospital / Primary Children’s Medical Center	Salt Lake City, UT	Non-author contributor	
Rachyl	Fisher		RN BSN	University of Utah Medical Center / Intermountain Medical Center / McKay-Dee Hospital / Utah Valley Hospital / Primary Children’s Medical Center	Salt Lake City, UT	Non-author contributor	
Susie	Solosth Moody		RN	University of Utah Medical Center / Intermountain Medical Center / McKay-Dee Hospital / Utah Valley Hospital / Primary Children’s Medical Center	Salt Lake City, UT	Non-author contributor	

Supplemental Online Content: Nonauthor Collaborators
*First name, last name, and suffix (if applicable) are required and will appear in PubMed.

***First Name and Middle Initial(s)**	***Last Name**	***Suffix (eg, Jr, III)**	Academic Degrees	Institution	Location (city, state/province, country)	Role or Contribution, eg, chair, principal investigator	Group (if more than 1 Group listed in the byline) and/or Subgroup (eg, Steering Committee)
Seetha	Shankaran		MD	Wayne State University / Hutzel Women's Hospital / Children's Hospital of Michigan	Detroit, MI	Non-author contributor	
Sanjay	Chawla		MD	Wayne State University / Hutzel Women's Hospital / Children's Hospital of Michigan	Detroit, MI	Non-author contributor	
Rebecca	Bara		RN	Wayne State University / Hutzel Women's Hospital / Children's Hospital of Michigan	Detroit, MI	Non-author contributor	
Mary E.	Johnson		RN	Wayne State University / Hutzel Women's Hospital / Children's Hospital of Michigan	Detroit, MI	Non-author contributor	
John	Barks		MD	Wayne State University / Hutzel Women's Hospital / Children's Hospital of Michigan	Detroit, MI	Non-author contributor	
Mary K.	Christensen		BA RRT	Wayne State University / Hutzel Women's Hospital / Children's Hospital of Michigan	Detroit, MI	Non-author contributor	
Diane F.	White		RRT CCRP	Wayne State University / Hutzel Women's Hospital / Children's Hospital of Michigan	Detroit, MI	Non-author contributor	

Supplemental Online Content: Nonauthor Collaborators
*First name, last name, and suffix (if applicable) are required and will appear in PubMed.

***First Name and Middle Initial(s)**	***Last Name**	***Suffix (eg, Jr, III)**	Academic Degrees	Institution	Location (city, state/province, country)	Role or Contribution, eg, chair, principal investigator	Group (if more than 1 Group listed in the byline) and/or Subgroup (eg, Steering Committee)
Stephanie A.	Wiggins		MS	Wayne State University / Hutzel Women's Hospital / Children's Hospital of Michigan	Detroit, MI	Non-author contributor	
