# Supplementary material for: Time to Full Enteral Feeds and Late-Onset Sepsis in Extremely Preterm Infants
Source: JAMA Netw Open. 2025 Nov 17;8(11):e2543940. doi: 10.1001/jamanetworkopen.2025.43940 (PMC12625685; doi:10.1001/jamanetworkopen.2025.43940)
Supplement: Supplement 3. — Data Sharing Statement [file jamanetwopen-e2543940-s003.pdf]

Data Sharing Statement

Salas. Time to Full Enteral Feeds and Late-Onset Sepsis in Extremely Preterm Infants. *JAMA Netw Open*. Published November 17, 2025. doi:10.1001/jamanetworkopen.2025.43940

Data
Data available: Yes
Data types: Deidentified participant data
How to access data: asalas@uab.edu
When available: With publication

Supporting Documents
Document types: None

Additional Information
Who can access the data: researchers whose proposed use of the data has been approved
Types of analyses: for a specified purpose
Mechanisms of data availability: Signed data access agreement
